# Supplementary material for: Theoretical analysis reveals a role for RAF conformational autoinhibition in paradoxical activation
Source: eLife. 2023 Oct 12;12:e82739. doi: 10.7554/eLife.82739 (PMC10627510; doi:10.7554/eLife.82739)
Supplement: Source code 1. — Contents include a folder that includes Mathematica notebooks that derive the analytic results. Contents also include a folder that includes Python notebooks that reproduce all of the numerical results. [file elife-82739-code1.zip › Supplementary_code/Analytical_Validations_Mathematica_All_Models/Mathematica_AllModels_FullFile.pdf]

```
$Version (*Mathematica version used to generate this file on April 14 2023.*)
```

Out[ ]= 12.0.0 for Microsoft Windows (64-bit) (April 6, 2019)

1. Distinct Mechanisms of PA

```
In[1]:= baseparams = {KA → 10, Kdim → 0.1, RAF → 0.04, f → 0.01, g → 100};
(*units: μM ∀ params but KA*)
```

Section 1.1. Dimer Potentiation (DP) Model

```
In[ ]:= Quit[]
```

1.1.1. Analytic Solution to the model

The assembled kinase state can dimerize (AA) or bind with a drug (Ad). The kinase dimer (AA), upon drug administration can occur in either partly (AAd) or fully inhibited state (AdAd). The equilibrium state relationships and protein concentration conservation equations for both total RAF (RAF) and total drug (DTOT) are defined as follows.

```
In[2]:= vars = {a, A, d, AA, Ad, AAd, AdAd};(*A list of all variables*)
rep1 = {AA → A^2/Kdim, AAd → 2 A^2 d/(f Kdim Kd), AdAd → A^2 d^2/(f g Kdim Kd^2), Ad → A d/Kd, a → 0} /. g → 1;
(*equations derived from equilibrium relationships*)
Consrv[eqns_] :=
 {Simplify[eqns[[1]] + eqns[[2]] + eqns[[5]] + 2 (eqns[[4]] + eqns[[6]] + eqns[[7]])],
  Simplify[eqns[[3]] + eqns[[5]] + eqns[[6]] + 2 (eqns[[7]])]}
  (*conservation relations for RAF and DTOT*)
eqnsconsrv = Simplify[Thread[Simplify[Consrv[vars] /. rep1] == {RAF, DTOT}]]
 (*Solve the conservation equations simplifying to two variables RAF,
 DTOT using the equilibrium relationships above*)
```

Out[4]= $$\left\{A\left(d+Kd+\frac{2\,A\left(d^2+2\,d\,Kd+f\,Kd^2\right)}{f\,Kd\,Kdim}\right)==Kd\,RAF,\ d+\frac{A\,d}{Kd}+\frac{2\,A^2\,d\,(d+Kd)}{f\,Kd^2\,Kdim}==DTOT\right\}$$

A simultaneous solution to both the above conservation equations is unwieldy and hard to obtain. Instead, we find partial solutions for kinase protomers as a function of free drug and then free drug as a function of the total drug and the kinase protomers. The latter solution is used to construct d vs DTOT relationship and prove monotonocity thereof leading to a complete solution to PA existence problem.

```
In[5]:= SimplifyPars[x_] := Simplify[x, {RAF > 0, DTOT > 0, Kd > 0, Kdim > 0, KA > 0, d > 0, f > 0, g > 0, d_rel > 0}];
sol1A = SimplifyPars[Solve[eqnsconsrv[[1]], A]][[2]] (*Second solution is positive*)
sol1d = SimplifyPars[Solve[eqnsconsrv[[2]], d]][[2]](*Second solution is positive*)
```

Out[6]= $$\left\{A\to\left(Kd\left(-d\,f\,Kdim-f\,Kd\,Kdim+\sqrt{\left(f\,Kdim\left(f\,Kd^2\,(Kdim+8\,RAF)+d^2\,(f\,Kdim+8\,RAF)+2\,d\,Kd\,(f\,Kdim+8\,RAF)\right)\right)}\right)\right)\Big/\left(4\left(d^2+2\,d\,Kd+f\,Kd^2\right)\right)\right\}$$

Out[7]= $$\left\{d\to-\frac{1}{4\,A^2}Kd\left(2\,A^2+A\,f\,Kdim+f\,Kd\,Kdim-\sqrt{\left(8\,A^2\,DTOT\,f\,Kdim+\left(2\,A^2+A\,f\,Kdim+f\,Kd\,Kdim\right)^2\right)}\right)\right\}$$

We estimate the total activity by counting all of the drug-free protomers which occur within a partly active or fully active dimer. Substituting the above solution into the expression for Raf activity and dividing by total RAF concentration, we obtain the proportionate activity as a function of total drug and other parameters.

In[8]:=
```
RafActivity[vars_] := 2 vars[[4]] + vars[[6]]
fnActiveRAFDS = SimplifyPars[RafActivity[vars] / RAF /. rep1 /. sol1A];
repratios = {RAF → Kdim RAF_rel, d → Kd d_rel};
fnActiveRAFDS = FullSimplify[fnActiveRAFDS /. repratios, {Kd > 0, Kdim > 0, RAF > 0}]
fnActiveRAF2DS =
 FullSimplify[SimplifyPars[fnActiveRAFDS //. {RAF_rel → E2d / (8 f (f + 2 d_rel + d_rel^2))}] /. d_rel → (E1d / f - 1),
  {E1d > 0, E2 > 0, f > 0}]
```

Out[11]= $\left((f + d_{rel}) \left(f + f\, d_{rel} - \sqrt{\left(f \left(f (1 + d_{rel})^2 + 8 (f + d_{rel} (2 + d_{rel}))\, RAF_{rel}\right)\right)}\right)^2\right) / \left(8 f (f + d_{rel} (2 + d_{rel}))^2\, RAF_{rel}\right)$

Out[12]= $$\frac{\left(E1d - \sqrt{E1d^2 + E2d}\right)^2 f (E1d + (-1 + f) f)}{E2d \left(E1d^2 + (-1 + f) f^2\right)}$$

Note that the above equations represent the total active RAF protomers in proportion to the total RAF kinase as a function of unbound drug (d). In order to analytically establish parameter values which correspond to activation of the kinase, we find the first derivative of the function fnActiveRAFDS and search for it's zeroes in section 1.1.3.

First, we identify total RAF dimers and it's proportion to active RAF dimers.

In[13]:=
```
RafDimers[eqns_] := (eqns[[4]] + eqns[[6]] + eqns[[7]]);
fnDimers = FullSimplify[SimplifyPars[(RafDimers[vars] / RAF /. rep1 /. sol1A) /. repratios], Kd > 0]
SimplifyPars[fnDimers / fnActiveRAFDS]
```

Out[14]= $\left(f + f\, d_{rel} - \sqrt{\left(f \left(f (1 + d_{rel})^2 + 8 (f + d_{rel} (2 + d_{rel}))\, RAF_{rel}\right)\right)}\right)^2 / \left(16 f (f + d_{rel} (2 + d_{rel}))\, RAF_{rel}\right)$

Out[15]= $$\frac{f + d_{rel} (2 + d_{rel})}{2 (f + d_{rel})}$$

- **1.1.2. Baseline Signaling (drug-free)**

In[16]:=
```
baselineActiveRAFDS = SimplifyPars[fnActiveRAFDS /. d_rel → 0]
FullSimplify[D[baselineActiveRAFDS, RAF_rel], RAF_rel > 0]
```

Out[16]= $$\frac{\left(-1 + \sqrt{1 + 8\, RAF_{rel}}\right)^2}{8\, RAF_{rel}}$$

Out[17]= $$\frac{\left(-1 + \sqrt{1 + 8\, RAF_{rel}}\right)^2}{8\, RAF_{rel}^2 \sqrt{1 + 8\, RAF_{rel}}}$$

The derivative of baseline signaling relative to RAF_{rel} is a positive definite function. Therefore, baseline signaling is a monotonically proportionate to RAF_{rel}, which is in turn, monotonically proportionate to RAF concentration and monotonically inverse relationship to Kdim.

Hence in the DS model, increasing RAF_{rel} increases baseline signaling. This is a simple analytic cross-check of expected behaviors in this model.

■ 1.1.3. Conditions on parameter regions for activation in response to the drug

In[18]:= **dfn12 = SimplifyPars[D[fnActiveRAFDS, d_{rel}]]**

Out[18]=
$$\frac{1}{8\, f\, (f + d_{rel}\, (2 + d_{rel}))^3\, RAF_{rel}} \left(f + f\, d_{rel} - \sqrt{\left(f \left(f\, (1 + d_{rel})^2 + 8\, (f + d_{rel}\, (2 + d_{rel}))\, RAF_{rel}\right)\right)}\right) \Bigg(2\, (f + d_{rel})\, (f + d_{rel}\, (2 + d_{rel})) \left(f - (f\, (1 + d_{rel})\, (f + 8\, RAF_{rel})) \Big/ \left(\sqrt{\left(f \left(f\, (1 + d_{rel})^2 + 8\, (f + d_{rel}\, (2 + d_{rel}))\, RAF_{rel}\right)\right)}\right)\right) - 4\, (1 + d_{rel})\, (f + d_{rel}) \left(f + f\, d_{rel} - \sqrt{\left(f \left(f\, (1 + d_{rel})^2 + 8\, (f + d_{rel}\, (2 + d_{rel}))\, RAF_{rel}\right)\right)}\right) + (f + d_{rel}\, (2 + d_{rel})) \left(f + f\, d_{rel} - \sqrt{\left(f \left(f\, (1 + d_{rel})^2 + 8\, (f + d_{rel}\, (2 + d_{rel}))\, RAF_{rel}\right)\right)}\right)\Bigg)$$

In[22]:= **existPADS = SimplifyPars[Reduce[dfn12 > 0]]**

Out[22]=
$$\Big(0 < RAF_{rel} < \left(f \left(f \left(3 - 8\, f + 4\, f^2\right) + (2 - 6\, f)\, d_{rel} - \left(1 + f + 4\, f^2\right) d_{rel}^2 - 4\, f\, d_{rel}^3 - d_{rel}^4\right)\right) \Big/ \left(8 \left(f + 2\, f\, d_{rel} + d_{rel}^2\right)^2\right) \;\&\&\; 2\, f + d_{rel} < 1 \;\&\&\; d_{rel} \le 1\Big) \;||\; \left(-\frac{f\, (1 + d_{rel})^2}{8 \left(f + 2\, d_{rel} + d_{rel}^2\right)} < RAF_{rel} < 0 \;\&\&\; f > 1\right) \;||\; \Big(\left(f \left(f \left(3 - 8\, f + 4\, f^2\right) + (2 - 6\, f)\, d_{rel} - \left(1 + f + 4\, f^2\right) d_{rel}^2 - 4\, f\, d_{rel}^3 - d_{rel}^4\right)\right) \Big/ \left(8 \left(f + 2\, f\, d_{rel} + d_{rel}^2\right)^2\right) < RAF_{rel} < 0 \;\&\&\; f \le 1 \;\&\&\; (d_{rel} > 1 \;||\; 2\, f + d_{rel} > 1)\Big)$$

Conditions for positive derivative of active RAF relative to unbound drug are shown above. However, RAF concentration is a positive definite quantity.

In[23]:= **existPADS = Simplify[existPADS, RAF_{rel} > 0]**

Out[23]=
$$RAF_{rel} < \left(f \left(f \left(3 - 8\, f + 4\, f^2\right) + (2 - 6\, f)\, d_{rel} - \left(1 + f + 4\, f^2\right) d_{rel}^2 - 4\, f\, d_{rel}^3 - d_{rel}^4\right)\right) \Big/ \left(8 \left(f + 2\, f\, d_{rel} + d_{rel}^2\right)^2\right) \;\&\&\; 2\, f + d_{rel} < 1 \;\&\&\; d_{rel} \le 1$$

The above condition is realizable only when, the second and third conditions are true: d<Kd(1-2f) and d<Kd. The latter condition is automatically true when former one is. Further, the former condition can only be true when 2f<1, i.e. f<0.5 instead of f<1: which one would naively expect.

The first condition can only be True when the RHS is positive, since LHS is always so.

In[24]:= **existPADS2 = SimplifyPars[Reduce[(existPADS[[1]][[2]] > 0)]]**

Out[24]=
$$(2\, f + d_{rel} < 1 \;\&\&\; d_{rel} \le 1) \;||\; f > \frac{1}{4}\left(3 + d_{rel} + 2\, d_{rel}^2 + (1 + d_{rel})\, \sqrt{9 + 4\, d_{rel} + 4\, d_{rel}^2}\right)$$

The first condition is identical to second and third conditions of existPADS which implies that f<0.5. The second alternate condition is f>3/4 + a positive number, which is a direct contradiction. Only one of these conditions can be satisfied. However, since the logic of existPADS equation is (A|B)&A the solution is A alone.

In[26]:= **existPADS[[2]]**

Out[26]= $2\, f + d_{rel} < 1$

Hence we derive the above, necessary but not sufficient condition for PA. It also suggests that for sufficiently small concentration of RAF and sufficiently small f, there is a small enough concentration of drug for which PA will be observed within this model.
Also, since d_{rel}<DTOT/Kd, $2\, f < 1 - d_{rel}$ is automatically satisfied if 2f<1-DTOT/Kd.
A corollary: with sufficiently high drug concentration, this condition cannot be satisfied. That is a validation that this model does not support motonic rise in activation in repsonse to the drug, eventually, the activity gets reduced.

Can PA exist even when above conditions are not satisfied? That is the first derivative of active RAF relative to drug is negative near zero drug but then becomes positive? And then negative again (since high drug conc have to go to zero)? - the possibility cannot be ruled out at this stage. This is why the conditions are necessary but not sufficient.

■ 1.1.4. Monotonic relationship between unbound (d) and total (DTOT) drug concentrations

For some of the conclusions with regards to PA to hold, the relationship between unbount and total drug has to be monotonic.

In[27]:=
```
eqnsconsrv[[2]]
```

Out[27]=

$$d + \frac{A\, d}{Kd} + \frac{2\, A^2\, d\, (d + Kd)}{f\, Kd^2\, Kdim} == DTOT$$

The derivative of the function DTOT relative to d is the sum of the derivative of different terms within the second equation in expression above. Among these the derivative of the first term is 1. And the derivative of the second and third term are shown as non-negative functions of unbound drug d, below.

In[31]:=
```
sd11 = FullSimplify[SimplifyPars[D[A*d /. sol1A, d]]];
rd11 = Reduce[sd11 < 0];
SimplifyPars[rd11]
```

Out[33]=
```
False
```

The first component is positive definite.

In[34]:=
```
sd12 = SimplifyPars[D[Simplify[A^2 d (d + Kd) /. sol1A], d]]
```

Out[34]=
```
              1
--------------------------------
16 (d^2 + 2 d Kd + f Kd^2)^3

Kd^2 (d f Kdim + f Kd Kdim - √(f Kdim (f Kd^2 (Kdim + 8 RAF) + d^2 (f Kdim + 8 RAF) + 2 d Kd (f Kdim + 8 RAF))))
  (2 d (d + Kd) (d^2 + 2 d Kd + f Kd^2) (f Kdim - (f (d + Kd) Kdim (f Kdim + 8 RAF)) /
        (√(f Kdim (f Kd^2 (Kdim + 8 RAF) + d^2 (f Kdim + 8 RAF) + 2 d Kd (f Kdim + 8 RAF))))) - 4 d (d + Kd)^2
      (d f Kdim + f Kd Kdim - √(f Kdim (f Kd^2 (Kdim + 8 RAF) + d^2 (f Kdim + 8 RAF) + 2 d Kd (f Kdim + 8 RAF)))) +
    d (d^2 + 2 d Kd + f Kd^2)
      (d f Kdim + f Kd Kdim - √(f Kdim (f Kd^2 (Kdim + 8 RAF) + d^2 (f Kdim + 8 RAF) + 2 d Kd (f Kdim + 8 RAF)))) +
    (d + Kd) (d^2 + 2 d Kd + f Kd^2)
      (d f Kdim + f Kd Kdim - √(f Kdim (f Kd^2 (Kdim + 8 RAF) + d^2 (f Kdim + 8 RAF) + 2 d Kd (f Kdim + 8 RAF)))))
```

Note that the first non-trivial product term in this expression is negative definite. This is because the term inside the square root is always greater than the term outside. So we focus on the second, larger term.

In[35]:=
```
sd121 = FullSimplify[sd12[[-1]]];
rd12 = Reduce[sd121 > 0];
SimplifyPars[rd12]
```

Out[37]=
```
False
```

Since the expression sd121 is never positive. Also, the coefficient to sd121 in sd12 is negative definite as stated above. Therefore the full expression sd12 which a product of the two is non negative for all positive values of the parameters.

DTOT=d+sd11+sd12

Since all three expressions on RHS are non-negative derivatives as a function of unbound drug - the total drug has a non-negative derivative. Therefore, DTOT(d) is monotonically positive/increasing function.
We exemplify this point numerically with an example set of parameters in the plot below.

In[38]:=
```
fnDTOT = SimplifyPars[eqnsconsrv[[2]][[1]] /. rep1 /. sol1A]
```

Out[38]=
```
d + (d (d + Kd) (d f Kdim + f Kd Kdim -
        √(f Kdim (f Kd^2 (Kdim + 8 RAF) + d^2 (f Kdim + 8 RAF) + 2 d Kd (f Kdim + 8 RAF))))^2) /
    (8 f (d^2 + 2 d Kd + f Kd^2)^2 Kdim) + (d (-d f Kdim - f Kd Kdim + √(f Kdim
          (f Kd^2 (Kdim + 8 RAF) + d^2 (f Kdim + 8 RAF) + 2 d Kd (f Kdim + 8 RAF))))) / (4 (d^2 + 2 d Kd + f Kd^2))
```

In[39]:=
```
Sty[x_] := Style[x, 20, FontFamily → "Arial"];
KTarr = {1. × 10^-6, 1. × 10^-7, 1. × 10^-8};
kdarr = {Kd → 1. × 10^-7 , Kd → 1. × 10^-9};
pltfn = Flatten[Table[fnDTOT /. kd, {kd, kdarr}, {RAF, KTarr}]] /. baseparams;
ps = {Blue, Green, Directive[Red, Thick], Directive[Blue, Dashed],
   Directive[Green, Dashed], Directive[Red, Dashed, Thick]};
LogLogPlot[pltfn, {d, 10^-9, 10^-5}, Frame → True, FrameTicksStyle → 20, PlotStyle → ps,
 PlotLegends → Placed[LineLegend[ {Blue, Green, Directive[Red, Thick]}, Sty /@ KTarr,
    LegendLabel → Sty["RAF (M)"], LegendFunction → Panel, LabelStyle → 12], {Right, Bottom}],
 FrameLabel → (Sty /@ {"Unbound Drug [d] (M)", "Total Drug [DTOT] (M)"}), ImageSize → {500},
 FrameStyle → Thickness[0.004], PlotLabel → Sty["Kd: —100nM  - -1nM KA:10"]
 (*N[TableForm[parset01,TableDirections→Row]]*)]
```

Out[44]=

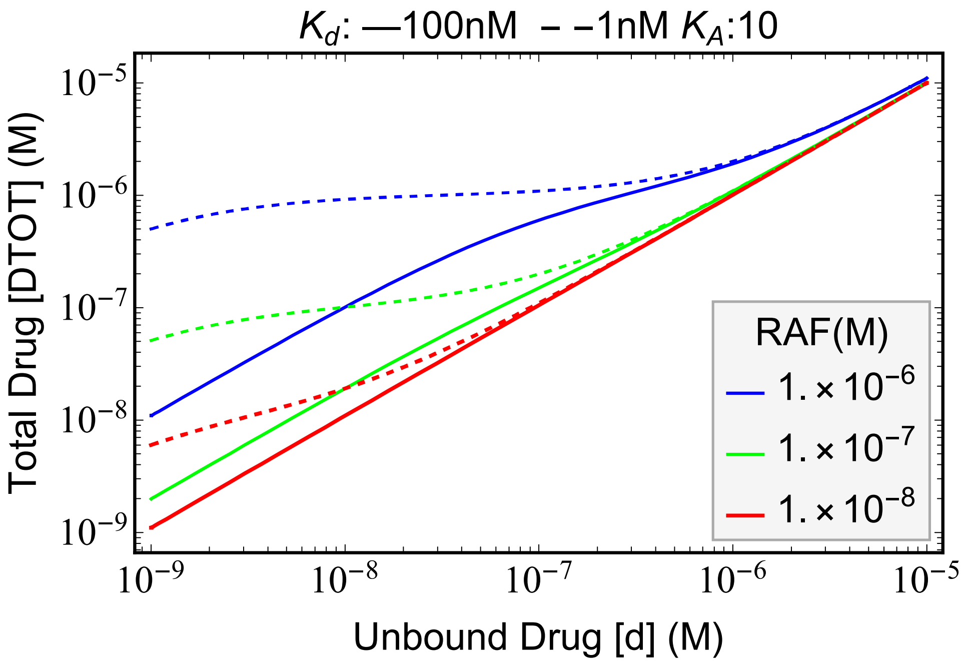

- **1.1.5. Analytic Expressions for maximum Fold Change (FC)**

Fold change is defined as the ratio between maximum RAF activity (active protomers) to that in absence of drug

In[45]:=
```
rafFC = SimplifyPars[fnActiveRAFDS / baselineActiveRAFDS]
```

Out[45]=

$$\left((f + d_{rel})\left(f + f\, d_{rel} - \sqrt{\left(f\left(f(1 + d_{rel})^2 + 8\,(f + d_{rel}(2 + d_{rel}))\, RAF_{rel}\right)\right)}\right)^2\right) \Big/ \left(f\,(f + d_{rel}(2 + d_{rel}))^2 \left(-1 + \sqrt{1 + 8\, RAF_{rel}}\right)^2\right)$$

Activating Range is defined as the lowest concentration above which the drug no longer acts as a paradoxical activator and becomes an inhibitor. This function is not easily solved analytically, and to understand its variation within the range of parameters defined in conditions for PA above, is calculated numerically.

Section 1.2. Negative Cooperativity (NC) Model

In[ ]:=
```
Quit[]
```

- **1.2.1. Analytic Solution to the model**

The assembled kinase state can dimerize (AA) or bind with a drug (Ad). The kinase dimer (AA), upon drug administration can occur in

either partly (AAd) or fully inhibited state (AdAd). The equilibrium state relationships and protein concentration conservation equations for both total RAF (RAF) and total drug (DTOT) are defined as follows.

In[46]:=
```
vars = {a, A, d, AA, Ad, AAd, AdAd}; (*A list of all variables*)
rep2 = {AA → A^2/Kdim, AAd → 2 A^2 d/(f Kdim Kd), AdAd → A^2 d^2/(f g Kdim Kd^2), Ad → A d/Kd, a → 0} /. f → 1;
(*derived from equilibrium relationships*)
Consrv[eqns_] :=
  {Simplify[eqns[[1]] + eqns[[2]] + eqns[[5]] + 2 (eqns[[4]] + eqns[[6]] + eqns[[7]])],
   Simplify[eqns[[3]] + eqns[[5]] + eqns[[6]] + 2 (eqns[[7]])]};
 (*conservation relations for RAF and DTOT*)
eqnsconsrv = Simplify[Thread[Simplify[Consrv[vars] /. rep2] == {RAF, DTOT}]]
```

Out[49]=

$$\left\{A\left(d+Kd+\frac{2\,A\left(d^2+2\,d\,g\,Kd+g\,Kd^2\right)}{g\,Kd\,Kdim}\right)==Kd\,RAF,\ d+\frac{A\,d}{Kd}+\frac{2\,A^2\,d\,(d+g\,Kd)}{g\,Kd^2\,Kdim}==DTOT\right\}$$

A simultaneous solution to both the above conservation equations is unwieldy and hard to obtain. Instead, we find partial solutions for kinase protomers as a function of free drug and then free drug as a function of the total drug and the kinase protomers. The latter solution is used to numerically construct d vs DTOT relationship.

In[50]:=
```
SimplifyPars[x_] :=
  Simplify[x, {RAF > 0, DTOT > 0, Kd > 0, Kdim > 0, KA > 0, d > 0, f > 0, g > 0, d_rel > 0, RAF_rel > 0}];
sol2A = SimplifyPars[Solve[eqnsconsrv[[1]], A]][[2]] (*Second solution is positive*)
sol2d = SimplifyPars[Solve[eqnsconsrv[[2]], d]][[2]](*Second solution is positive*)
```

Out[51]=

$$\left\{A\to -\left(\left(g\,Kd\,Kdim\left(d+Kd-\sqrt{\left(\frac{1}{g\,Kdim}\left(2\,d\,g\,Kd\,(Kdim+8\,RAF)+g\,Kd^2\,(Kdim+8\,RAF)+d^2\,(g\,Kdim+8\,RAF)\right)\right)}\right)\right)\Big/\left(4\left(d^2+2\,d\,g\,Kd+g\,Kd^2\right)\right)\right)\right\}$$

Out[52]=

$$\left\{d\to -\frac{1}{4\,A^2}g\,Kd\left(2\,A^2+A\,Kdim+Kd\,Kdim+\sqrt{\left(\frac{1}{g}\left(8\,A^2\,DTOT\,Kdim+g\left(2\,A^2+A\,Kdim+Kd\,Kdim\right)^2\right)\right)}\right)\right\}$$

We estimate the total activity by counting all of the drug-free protomers which occur within a partly active or fully active dimer. Substituting the above solution into the expression for Raf activity and dividing by total RAF concentration, we obtain the proportionate activity as a function of total drug and other parameters.

In[63]:=
```
RafActivity[vars_] := 2 vars[[4]] + vars[[6]]
fnActiveRAF = SimplifyPars[RafActivity[vars] / RAF /. rep2 /. sol2A];
repratios = {Kdim → RAF/RAF_rel, d → Kd d_rel};
fnActiveRAFNC = SimplifyPars[FullSimplify[fnActiveRAF /. repratios]]
fnActiveRAF2NC =
 FullSimplify[SimplifyPars[fnActiveRAFNC //. {(1 + d_rel) → E1n, RAF_rel → E2n/(g + 2 g d_rel + d_rel^2)/8}]]
```

Out[66]=

$$\left(g^2\,(1+d_{rel})\left(1+d_{rel}-\sqrt{\left((1+d_{rel})^2+\frac{8\left(g+2\,g\,d_{rel}+d_{rel}^2\right)RAF_{rel}}{g}\right)}\right)^2\right)\Big/\left(8\left(g+2\,g\,d_{rel}+d_{rel}^2\right)^2 RAF_{rel}\right)$$

Out[67]=

$$\frac{E1n\left(E1n-\sqrt{E1n^2+\frac{E2n}{g}}\right)^2 g^2}{E2n\left(g+2\,g\,d_{rel}+d_{rel}^2\right)}$$

Note that the above equations represent the total active RAF protomers in proportion to the total RAF kinase as a function of unbound drug

(d). In order to analytically establish parameter values which correspond to activation of the kinase, we find the first derivative of this function and search for it's zeroes.

In[68]:=
```
RafDimers[eqns_] := (eqns[[4]] + eqns[[6]] + eqns[[7]]);
fnDimersNC = FullSimplify[SimplifyPars[(RafDimers[vars] / RAF /. rep2 /. sol2A) /. repratios]]
SimplifyPars[fnDimersNC / fnActiveRAFNC]
```

Out[69]=
$$\left(g\left(1+d_{rel}-\sqrt{\left((1+d_{rel})^2+\frac{8\left(g+2\,g\,d_{rel}+d_{rel}^2\right)RAF_{rel}}{g}\right)}\right)^2\right)\Big/\left(16\left(g+2\,g\,d_{rel}+d_{rel}^2\right)RAF_{rel}\right)$$

Out[70]=
$$\frac{g+2\,g\,d_{rel}+d_{rel}^2}{2\,g+2\,g\,d_{rel}}$$

- **1.2.2. Baseline Signaling (drug-free)**

In[71]:=
```
baselineActiveRAFNC = fnActiveRAFNC /. d_rel → 0
SimplifyPars[D[baselineActiveRAFNC, RAF_rel]]
```

Out[71]=
$$\frac{\left(1-\sqrt{1+8\,RAF_{rel}}\right)^2}{8\,RAF_{rel}}$$

Out[72]=
$$\frac{\left(-1+\sqrt{1+8\,RAF_{rel}}\right)^2}{8\,RAF_{rel}^2\sqrt{1+8\,RAF_{rel}}}$$

The derivative of baseline signaling relative to RAF_{rel} is a positive definite function. Therefore, baseline signaling is a monotonically proportionate to RAF_{rel}.

- **1.2.3. Conditions on parameter regions for activation in response to the drug**

In[73]:=
```
dfn2 = SimplifyPars[D[fnActiveRAFNC, d_rel]]
```

Out[73]=
$$\frac{1}{8\left(g+2\,g\,d_{rel}+d_{rel}^2\right)^3RAF_{rel}}g^2\left(1+d_{rel}-\sqrt{(1+d_{rel})^2+\frac{8\left(g+2\,g\,d_{rel}+d_{rel}^2\right)RAF_{rel}}{g}}\right)$$
$$\left(-4\,(1+d_{rel})\,(g+d_{rel})\left(1+d_{rel}-\sqrt{\left((1+d_{rel})^2+\frac{8\left(g+2\,g\,d_{rel}+d_{rel}^2\right)RAF_{rel}}{g}\right)}\right)+\right.$$
$$\left(g+2\,g\,d_{rel}+d_{rel}^2\right)\left(1+d_{rel}-\sqrt{\left((1+d_{rel})^2+\frac{8\left(g+2\,g\,d_{rel}+d_{rel}^2\right)RAF_{rel}}{g}\right)}\right)+$$
$$\left.2\,(1+d_{rel})\left(g+2\,g\,d_{rel}+d_{rel}^2\right)\left(1+\frac{-g\,(1+8\,RAF_{rel})-d_{rel}\,(g+8\,RAF_{rel})}{g\sqrt{(1+d_{rel})^2+\frac{8\left(g+2\,g\,d_{rel}+d_{rel}^2\right)RAF_{rel}}{g}}}\right)\right)$$

In[74]:=
```
existPANC = SimplifyPars[Reduce[(dfn2 > 0)]]
```

Out[74]=
```
False
```

Therefore, the derivative of active RAF relative to unbound drug may never be positive, independent of the drug concentration and negative cooperativity. **This mechanism does not produce PA**.

1.2.5. Analytic Expressions for maximum Fold Change (FC)

Fold change is defined as the ratio between maximum RAF activity (no/conc of active protomers) to that in absence of drug

```
In[75]:= maxActiveRAFNC = FullSimplify[SimplifyPars[fnActiveRAFNC]];
         rafFC = SimplifyPars[maxActiveRAFNC / baselineActiveRAFNC]
```

Out[76]=
$$\left(g^2\,(1+d_{rel})\left(1+d_{rel}-\sqrt{(1+d_{rel})^2+\frac{8\left(g+2\,g\,d_{rel}+d_{rel}^2\right)RAF_{rel}}{g}}\right)^2\right)\Big/\left(\left(g+2\,g\,d_{rel}+d_{rel}^2\right)^2\left(-1+\sqrt{1+8\,RAF_{rel}}\right)^2\right)$$

```
In[77]:= dRAFrafFC = SimplifyPars[D[rafFC, RAF_rel]];
         dgrafFC = SimplifyPars[D[rafFC, g]];
         SimplifyPars[Reduce[dRAFrafFC > 0]]
         SimplifyPars[Reduce[dgrafFC > 0]]
```

Out[79]= $g > 1$

Out[80]= True

Hence, first derivative of the fold change expression relative to RAF_{rel} is always positive in the negative cooperativity model and always negative in positive cooperativity model. That is when g>1: negative cooperativity mediates increase in activity with increase in [RAF].

Even though fold change is not paradoxical (PA<1), as negative cooperativity increases, fold change increases. In other mechanisms where PA exists, negative cooperativity increases PA further.

Section 1.3. Conformal Autoinhibition (CA/base) Model

```
In[ ]:= Quit[]
```

1.3.1. Analytic Solution to the model

This model incorporates all the mechanisms from the part models to combine into a model of kinase which auto-inhibits (a), which, for simplicity, is modeled to relieve at a first order rate to an assembled state (A). The assembled kinase state can dimerize (AA) or bind with a drug (Ad). The kinase dimer (AA), upon drug administration can occur in either partly (AAd) or fully inhibited state (AdAd). The equilibrium state relationships and protein concentration conservation equations for both total RAF (RAF) and total drug (DTOT) are defined as follows.

```
In[81]:= vars = {a, A, d, AA, Ad, AAd, AdAd};(*A list of all variables*)
         rep3 = {AA -> A^2/Kdim, AAd -> 2 A^2 d/(f Kdim Kd), AdAd -> A^2 d^2/(f g Kdim Kd^2), Ad -> A d/Kd, a -> A KA} /. {f -> 1, g -> 1};
         (*derived from equilibrium relationships*)
         Consrv[eqns_] :=
           {Simplify[eqns[[1]] + eqns[[2]] + eqns[[5]] + 2 (eqns[[4]] + eqns[[6]] + eqns[[7]])],
            Simplify[eqns[[3]] + eqns[[5]] + eqns[[6]] + 2 (eqns[[7]])]};
          (*conservation relations for RAF and DTOT*)
         eqnsconsrv = Simplify[Thread[Simplify[Consrv[vars] /. rep3] == {RAF, DTOT}]]
```

Out[84]=
$$\left\{\frac{A\left(2\,A\,(d+Kd)^2+Kd\,(d+Kd+KA\,Kd)\,Kdim\right)}{Kd^2\,Kdim}==RAF,\ d+\frac{A\,d}{Kd}+\frac{2\,A^2\,d\,(d+Kd)}{Kd^2\,Kdim}==DTOT\right\}$$

A simultaneous solution to both the above conservation equations is unwieldy and hard to obtain. Instead, we find partial solutions for kinase protomers as a function of free drug and then free drug as a function of the total drug and the kinase protomers. The latter solution is used to numerically construct d vs DTOT relationship.

```
In[85]:= SimplifyPars[x_] := Simplify[x, {RAF > 0, DTOT > 0, Kd > 0, Kdim > 0, KA > 0, d > 0, f > 0, g > 0}];
sol3A = SimplifyPars[Solve[eqnsconsrv[[1]], A]][[2]] (*Second solution is positive*)
sol3d = SimplifyPars[Solve[eqnsconsrv[[2]], d]][[2]](*Second solution is positive*)
```

Out[86]= $\left\{A \to -\frac{1}{4\,(d+Kd)^2} Kd\, Kdim \left(d + Kd + KA\, Kd - \sqrt{\frac{(d + Kd + KA\, Kd)^2\, Kdim + 8\,(d+Kd)^2\, RAF}{Kdim}}\right)\right\}$

Out[87]= $\left\{d \to -\frac{1}{4\,A^2} Kd \left(2\,A^2 + A\, Kdim + Kd\, Kdim - \sqrt{\left(8\,A^2\, DTOT\, Kdim + \left(2\,A^2 + A\, Kdim + Kd\, Kdim\right)^2\right)}\right)\right\}$

We estimate the total activity by counting all of the drug-free protomers which occur within a partly active or fully active dimer. Substituting the above solution into the expression for Raf activity and dividing by total RAF concentration, we obtain the proportionate activity as a function of total drug and other parameters.

```
In[88]:= RafActivity[vars_] := 2 vars[[4]] + vars[[6]]
fnActiveRAF = SimplifyPars[RafActivity[vars] / RAF /. rep3 /. sol3A];
repratios = {Kdim → RAF/RAF_rel, d → Kd d_rel};
fnActiveRAFCA = SimplifyPars[FullSimplify[fnActiveRAF /. repratios]]
fnActiveRAF2CA = FullSimplify[
  SimplifyPars[fnActiveRAFCA //. {(1 + KA + d_rel) → E1c, RAF_rel → E2c / (1 + 2 d_rel + d_rel^2) / 8}]]
```

Out[91]= $\left(1 + KA + d_{rel} - \sqrt{(1 + KA + d_{rel})^2 + 8\,(1 + d_{rel})^2\, RAF_{rel}}\right)^2 \Big/ \left(8\,(1 + d_{rel})^3\, RAF_{rel}\right)$

Out[92]= $\frac{\left(E1c - \sqrt{E1c^2 + E2c}\right)^2}{E2c\,(1 + d_{rel})}$

Note that the above equations represent the total active RAF protomers in proportion to the total RAF kinase as a function of unbound drug (d). In order to analytically establish parameter values which correspond to activation of the kinase, we find the first derivative of this function (equation 11) and search for it's zeroes (equation 12).

```
In[93]:= RafDimers[eqns_] := (eqns[[4]] + eqns[[6]] + eqns[[7]]);
fnDimersCA = FullSimplify[SimplifyPars[(RafDimers[vars] / RAF /. rep3 /. sol3A) /. repratios]]
SimplifyPars[fnDimersCA / fnActiveRAFCA]
```

Out[94]= $\left(1 + KA + d_{rel} - \sqrt{(1 + KA + d_{rel})^2 + 8\,(1 + d_{rel})^2\, RAF_{rel}}\right)^2 \Big/ \left(16\,(1 + d_{rel})^2\, RAF_{rel}\right)$

Out[95]= $\frac{1}{2}\,(1 + d_{rel})$

- **1.3.2. Baseline Signaling (drug-free)**

```
In[96]:= baselineActiveRAFCA = fnActiveRAFCA /. d_rel → 0;
rep3b = {RAF_rel → E3 (1 + KA)^2/8};
baselineActiveRAF1 = SimplifyPars[baselineActiveRAFCA /. rep3b]
SimplifyPars[D[baselineActiveRAF1, E3] /. rep3b]
```

Out[98]= $\frac{\left(-1 + \sqrt{1 + E3}\right)^2}{E3}$

Out[99]= $\frac{\left(-1 + \sqrt{1 + E3}\right)^2}{E3^2\,\sqrt{1 + E3}}$

The derivative of baseline signaling relative to E3 is a positive definite function. Therefore, baseline signaling is a monotonically proportionate to E3, which is in turn, monotonically proportionate to RAF concentration and monotonically inverse relationship to KA.

Hence in the base model, increasing KA decreases baseline signaling and increasing RAF_{rel} increases baseline signaling.

■ 1.3.3. Conditions on parameter regions for activation in response to the drug

In[100]:= `dfn3 = SimplifyPars[D[fnActiveRAFCA, d_rel]]`

Out[100]=
$$\left(\left(1+KA+d_{rel}-\sqrt{(1+KA+d_{rel})^2+8\,(1+d_{rel})^2\,RAF_{rel}}\right)\left(2\,(1+d_{rel})\left(1-(1+KA+d_{rel}+8\,(1+d_{rel})\,RAF_{rel})\Big/\left(\sqrt{\left((1+KA+d_{rel})^2+8\,(1+d_{rel})^2\,RAF_{rel}\right)}\right)\right)-3\left(1+KA+d_{rel}-\sqrt{(1+KA+d_{rel})^2+8\,(1+d_{rel})^2\,RAF_{rel}}\right)\right)\right)\Big/\left(8\,(1+d_{rel})^4\,RAF_{rel}\right)$$

In[101]:= `Simplify[SimplifyPars[dfn3 //. {(1 + KA + d_rel) → E1, RAF_rel → E2 / (1 + 2 d_rel + d_rel^2) / 8}], {E1 > 0, E2 > 0, d_rel > 0}]`

Out[101]=
$$-\left(\left(\left(-E1+\sqrt{E1^2+E2}\right)\left(3\,E1^2+E2+2\sqrt{E1^2+E2}-E1\left(2+3\sqrt{E1^2+E2}\right)+2\left(-E1+\sqrt{E1^2+E2}\right)d_{rel}\right)\right)\Big/\left(E2\sqrt{E1^2+E2}\,(1+d_{rel})^2\right)\right)$$

The first two roots are complex coming from the first bracket term.

In[102]:= `zeroes = SimplifyPars[Solve[dfn3 == 0, d_rel, VerifySolutions → True]]`

Out[102]=
$$\left\{\left\{d_{rel}\to-\frac{1+KA+8\,RAF_{rel}+2\,KA\sqrt{1+6\,RAF_{rel}}}{1+8\,RAF_{rel}}\right\},\left\{d_{rel}\to-\frac{1+KA+8\,RAF_{rel}-2\,KA\sqrt{1+6\,RAF_{rel}}}{1+8\,RAF_{rel}}\right\}\right\}$$

The first solution is negative definite. Below, we derive the rules (expression 13) which allow the second solution to be positive

In[103]:= `exist = Reduce[((d_rel /. zeroes[[2]]) > 0) && (KA > 0) && (RAF_rel > 0)]`

Out[103]=
$$KA>1\ \&\&\ 0<RAF_{rel}<\frac{1}{8}\left(-1-2\,KA+3\,KA^2\right)$$

Hence with a sufficiently small ratio of raf concentration to dimerization constant ratio, the auto-inhibition mechanism can by itself mediate activation of the kinase in response to drug administration.

In section 1.2 we will show that our analytic results as a function of unbound drug concentration also extend to the complete case where we consider the relationship between unbound and bound drug.

To establish that the above constraints imply existence of activation (maxima), we derive the second derivative of fnActiveRAFCA, find it’s value at the critical point in variable ‘zeroes’. We then find the conditions under which this function is negative.

In[104]:= `d2fn3 = SimplifyPars[D[dfn3, d_rel]]`

Out[104]=
$$\frac{1}{8\,(1+d_{rel})^5\,RAF_{rel}}\Bigg((1+d_{rel})\left(1+KA+d_{rel}-\sqrt{(1+KA+d_{rel})^2+8\,(1+d_{rel})^2\,RAF_{rel}}\right)\left(-1-\frac{16\,KA^2\,(1+d_{rel})\,RAF_{rel}}{\left((1+KA+d_{rel})^2+8\,(1+d_{rel})^2\,RAF_{rel}\right)^{3/2}}-\frac{2\,(1+KA+d_{rel}+8\,(1+d_{rel})\,RAF_{rel})}{\sqrt{(1+KA+d_{rel})^2+8\,(1+d_{rel})^2\,RAF_{rel}}}+\frac{3\,(1+KA+8\,RAF_{rel}+d_{rel}\,(1+8\,RAF_{rel}))}{\sqrt{(1+KA+d_{rel})^2+8\,(1+d_{rel})^2\,RAF_{rel}}}\right)+(1+d_{rel})\left(1-\frac{1+KA+d_{rel}+8\,(1+d_{rel})\,RAF_{rel}}{\sqrt{(1+KA+d_{rel})^2+8\,(1+d_{rel})^2\,RAF_{rel}}}\right)\left(2\,(1+d_{rel})\left(1-(1+KA+d_{rel}+8\,(1+d_{rel})\,RAF_{rel})\Big/\left(\sqrt{\left((1+KA+d_{rel})^2+8\,(1+d_{rel})^2\,RAF_{rel}\right)}\right)\right)-3\left(1+KA+d_{rel}-\sqrt{(1+KA+d_{rel})^2+8\,(1+d_{rel})^2\,RAF_{rel}}\right)\right)-4\left(1+KA+d_{rel}-\sqrt{(1+KA+d_{rel})^2+8\,(1+d_{rel})^2\,RAF_{rel}}\right)\left(2\,(1+d_{rel})\left(1-(1+KA+d_{rel}+8\,(1+d_{rel})\,RAF_{rel})\Big/\left(\sqrt{\left((1+KA+d_{rel})^2+8\,(1+d_{rel})^2\,RAF_{rel}\right)}\right)\right)-3\left(1+KA+d_{rel}-\sqrt{(1+KA+d_{rel})^2+8\,(1+d_{rel})^2\,RAF_{rel}}\right)\right)\Bigg)$$

In[105]:= `d2fn3z = FullSimplify[d2fn3 /. zeroes[[2]], {KA > 0, RAF_rel > 0}]`

Out[105]= $\left(1-\sqrt{1+6\,RAF_{rel}}+3\,RAF_{rel}\left(6-5\sqrt{1+6\,RAF_{rel}}-24\,RAF_{rel}\left(-1+\sqrt{1+6\,RAF_{rel}}\right)\right)\right)\Big/\left(162\,KA^3\,RAF_{rel}\right)$

In[106]:= `Reduce[(d2fn3z < 0) && (KA >= 0) && (RAF_rel >= 0)]`

Out[106]= $KA > 0\ \&\&\ RAF_{rel} > 0$

No additional constraints from second derivative condition that the critical point be a maxima. The ‘exist’ condition above is necessary and sufficient as long as monotonocity between unbound and total drug can be established.

▪ 1.3.4. Monotonic relationship between unbound (d) and total (DTOT) drug concentrations

The derivative of the function DTOT relative to unbound drug d is the sum of the derivative of different composing terms. Among these the derivative of the first term is 1. And the derivative of the second and third term are shown as non-negative functions of unbound drug d, below.

In[122]:=
```
sd311 = FullSimplify[SimplifyPars[D[A*d /. sol3A, d]]];
rd311 = Reduce[sd311 < 0];
SimplifyPars[rd311]
```

Out[124]= $(3\,d \le Kd\ ||\ d < Kd)\ \&\&\ \left(d < Kd < 3\,d\ ||\ KA < \frac{d+Kd}{d-Kd}\ ||\ Kd > 3\,d\right)\ \&\&\ \left(\frac{d+Kd}{d-Kd} < KA < \frac{Kd}{d-Kd}\ ||\ KA < \frac{d+Kd}{d-Kd}\right)\ \&\&\ d\left(Kdim - KA^2\,Kdim + 8\,RAF\right) + Kd\left((1+KA)^2\,Kdim + 8\,RAF\right) < 0$

In[125]:=
```
sd312 = SimplifyPars[D[Simplify[A^2 d (d + Kd) /. sol3A], d]]
```

Out[125]=
$$\frac{1}{16\,(d+Kd)^4}Kd^2\,Kdim^2\left(d+Kd+KA\,Kd-\sqrt{\frac{(d+Kd+KA\,Kd)^2\,Kdim+8\,(d+Kd)^2\,RAF}{Kdim}}\right)\left(-3\,d\left(d+Kd+KA\,Kd-\sqrt{\frac{(d+Kd+KA\,Kd)^2\,Kdim+8\,(d+Kd)^2\,RAF}{Kdim}}\right)+(d+Kd)\left(d+Kd+KA\,Kd-\sqrt{\frac{(d+Kd+KA\,Kd)^2\,Kdim+8\,(d+Kd)^2\,RAF}{Kdim}}\right)+2\,d\,(d+Kd)\left(1+(-d\,(Kdim+8\,RAF)-Kd\,(Kdim+KA\,Kdim+8\,RAF))\Big/\left(\sqrt{\left(Kdim\left((d+Kd+KA\,Kd)^2\,Kdim+8\,(d+Kd)^2\,RAF\right)\right)}\right)\right)\right)$$

Note that the first non-trivial product term in this expression is negative definite as the term inside the square root is always greater than the term outside. So we focus on the second term.

In[126]:=
```
sd3121 = FullSimplify[sd312[[-1]]];
rd312 = Reduce[sd3121 > 0];
SimplifyPars[SimplifyPars[rd312]]
```

Out[128]=
$$Kd>2\,d\;\&\&\;\left(\frac{d+Kd}{2\,d-Kd}<KA<-\frac{Kd\,(d+Kd)}{-4\,d^2+Kd^2}\;||\;KA<\frac{d+Kd}{2\,d-Kd}\right)\;\&\&\;\frac{1}{8\,(d+Kd)^2}\left(d^2\left(1-4\,KA^2\right)+2\,d\,(1+KA)\,Kd+(1+KA)^2\,Kd^2\right)Kdim+RAF<0$$

The first two conditions imply: d<Kd/2 combined with KA<~1/(2d-Kd) which gives KA<0. As KA is positive definite, the expression sd3121 is non positive. Also, the coefficient to sd3121 in sd312 is negative definite as stated above. Therefore the full expression sd12 is non negative for all positive values of the parameters.

DTOT=d+sd11+sd12

Since all three expressions on RHS are non-negative derivatives as a function of unbound drug - the total drug has a non-negative derivative. Therefore, DTOT(d) is monotonically positive/increasing function.
We show this point with an example set of parameters in the plot below.

In[129]:=
```
fnDTOT = SimplifyPars[Consrv[vars][[2]] /. rep3 /. sol3A];
```

In[130]:=
```
Sty[x_] := Style[x, 20, FontFamily → "Arial"];
parset01 = {KA → 2., Kdim → 1. × 10^-7 (*100nM*), f → 0.01, g → 4};
KTarr = {1. × 10^-6, 1. × 10^-7, 1. × 10^-8};
kdarr = {Kd → 1. × 10^-7 , Kd → 1. × 10^-9};
pltfn = Flatten[Table[fnDTOT /. parset01 /. kd, {kd, kdarr}, {RAF, KTarr}]];
ps = {Blue, Green, Directive[Red, Thick], Directive[Blue, Dashed],
   Directive[Green, Dashed], Directive[Red, Dashed, Thick]};
LogLogPlot[pltfn, {d, 10^-9, 10^-5}, Frame → True, FrameTicksStyle → 20, PlotStyle → ps,
 PlotLegends → Placed[LineLegend[ {Blue, Green, Directive[Red, Thick]}, Sty /@ KTarr,
    LegendLabel → Sty["RAF (M) "], LegendFunction → Panel, LabelStyle → 12], {Right, Bottom}],
 FrameLabel → (Sty /@ {"Unbound Drug  [d]  (M)", "Total Drug  [DTOT]  (M)"}), ImageSize → {500},
 FrameStyle → Thickness[0.004], PlotLabel → Sty["K_d: —100nM  – –1nM K_A:2"]
 (*N[TableForm[parset01,TableDirections→Row]]*)]
```

Out[136]=

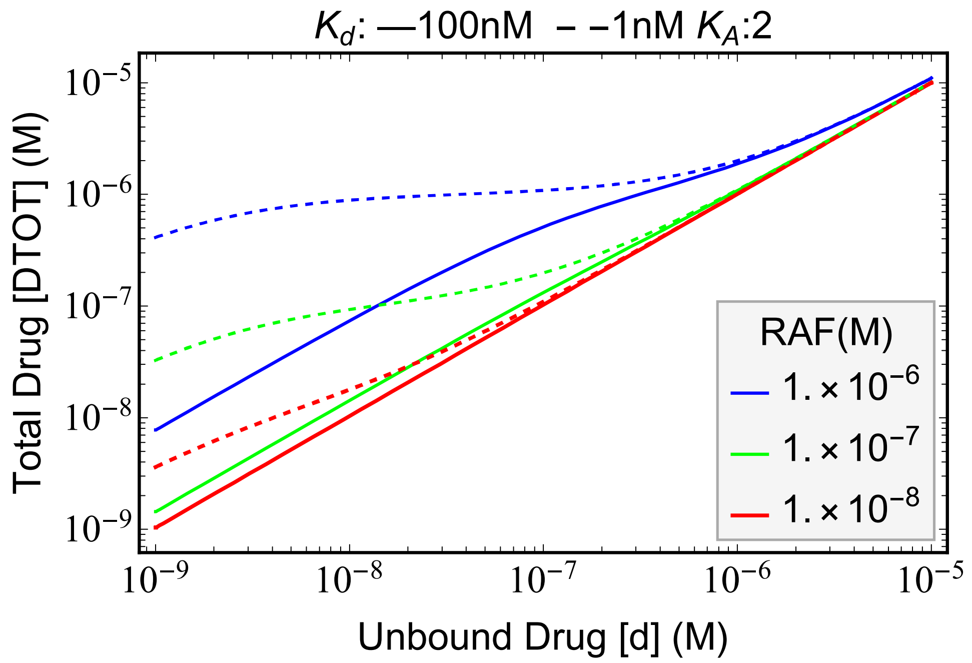

- **1.3.5. Analytic Expressions for maximum Fold Change (FC)**

Fold change is defined as the ratio between maximum RAF activity (no/conc of active protomers) to that in absence of drug

In[137]:=
```
maxActiveRAFCA = FullSimplify[SimplifyPars[fnActiveRAFCA /. zeroes[[2]]]];
rafFC = SimplifyPars[maxActiveRAFCA / baselineActiveRAFCA]
```

Out[138]=

$$\left(8\left(-1+\sqrt{1+6\,RAF_{rel}}+RAF_{rel}\left(-9+6\sqrt{1+6\,RAF_{rel}}\right)\right)\right)\Big/\left(27\,KA\left(1+KA-\sqrt{(1+KA)^2+8\,RAF_{rel}}\right)^2\right)$$

When the ‘exist’ conditions derived above to qualify for existence of PA within this model are satisfied, we can evaluate the functional dependence of the raf fold change expression on RAF_{rel} and KA:

In[139]:=
```
dRAFrafFC = SimplifyPars[D[rafFC, RAFrel]]
dKArafFC = SimplifyPars[D[rafFC, KA]]
Simplify[Reduce[dRAFrafFC < 0], exist]
Simplify[Reduce[dKArafFC > 0], exist]
```

Out[139]= $\left(8\left(\left(9\left(-1-6\,RAF_{rel}+\sqrt{1+6\,RAF_{rel}}\right)\left(-1-KA+\sqrt{(1+KA)^2+8\,RAF_{rel}}\right)\right)\middle/\left(\sqrt{1+6\,RAF_{rel}}\right)+\left(8\left(-1+\sqrt{1+6\,RAF_{rel}}+RAF_{rel}\left(-9+6\sqrt{1+6\,RAF_{rel}}\right)\right)\right)\middle/\left(\sqrt{(1+KA)^2+8\,RAF_{rel}}\right)\right)\right)\middle/\left(27\,KA\left(1+KA-\sqrt{(1+KA)^2+8\,RAF_{rel}}\right)^3\right)$

Out[140]= $-\left(\left(8\left(-2\,KA+\sqrt{(1+KA)^2+8\,RAF_{rel}}\right)\left(-1+\sqrt{1+6\,RAF_{rel}}+RAF_{rel}\left(-9+6\sqrt{1+6\,RAF_{rel}}\right)\right)\right)\middle/\left(27\,KA^2\sqrt{(1+KA)^2+8\,RAF_{rel}}\left(1+KA-\sqrt{(1+KA)^2+8\,RAF_{rel}}\right)^2\right)\right)$

Out[141]= True

Out[142]= True

Hence, first derivative of the fold change expression relative to KA is always positive when the model displays PA and the first derivative relative to RAF rel always negative.
In other words, an equilibrium that more and more favors the inactive state (increasing KA), the fold change continues to increase as a function of KA. And as the concentration of RAF increases or it’s dimerization rate increases (Kdim reduces), the fold change relative to baseline continues to reduce.

In[143]:=
```
{maxActiveRAFCA, baselineActiveRAFCA}
```

Out[143]= $\left\{\left(-1+\sqrt{1+6\,RAF_{rel}}+RAF_{rel}\left(-9+6\sqrt{1+6\,RAF_{rel}}\right)\right)\middle/\left(27\,KA\,RAF_{rel}\right),\ \frac{\left(1+KA-\sqrt{(1+KA)^2+8\,RAF_{rel}}\right)^2}{8\,RAF_{rel}}\right\}$

Activating Range is defined as the lowest concentration above which the drug no longer acts as a paradoxical activator and becomes an inhibitor. This function is not easily solved analytically, and to understand its variation within the range of parameters defined in conditions for PA above, is calculated numerically in python files.

Section 1.4. Unified Model

In[ ]:=
```
Quit[]
```

- **1.4.1. Analytic Solution to the model**

This model incorporates all the mechanisms.

In[144]:=
```
vars = {a, A, d, AA, Ad, AAd, AdAd};(*A list of all variables*)
rep4 = {AA → A^2/Kdim, AAd → 2 A^2 d/(f Kdim Kd), AdAd → A^2 d^2/(f g Kdim Kd^2), Ad → A d/Kd, a → A KA};
(*derived from equilibrium relationships*)
Consrv[eqns_] :=
  {Simplify[eqns[[1]] + eqns[[2]] + eqns[[5]] + 2 (eqns[[4]] + eqns[[6]] + eqns[[7]])],
   Simplify[eqns[[3]] + eqns[[5]] + eqns[[6]] + 2 (eqns[[7]])]};
 (*conservation relations for RAF and DTOT*)
eqnsconsrv = Simplify[Thread[Simplify[Consrv[vars] /. rep4] == {RAF, DTOT}]]
```

Out[147]=
$$\left\{A\left(d + Kd + KA\,Kd + \frac{2\,A\left(d^2 + 2\,d\,g\,Kd + f\,g\,Kd^2\right)}{f\,g\,Kd\,Kdim}\right) == Kd\,RAF,\ d + \frac{A\,d}{Kd} + \frac{2\,A^2\,d\,(d + g\,Kd)}{f\,g\,Kd^2\,Kdim} == DTOT\right\}$$

A simultaneous solution to both the above conservation equations is unwieldy and hard to obtain. Instead, we find partial solutions for kinase protomers as a function of free drug and then free drug as a function of the total drug and the kinase protomers. The latter solution is used to numerically construct d vs DTOT relationship.

In[148]:=
```
SimplifyPars[x_] :=
  Simplify[x, {RAF > 0, DTOT > 0, Kd > 0, Kdim > 0, KA > 0, d > 0, f > 0, g > 0, d_rel > 0, RAF_rel > 0}];
sol4A = SimplifyPars[Solve[eqnsconsrv[[1]], A]][[2]] (*Second solution is positive*)
sol4d = SimplifyPars[Solve[eqnsconsrv[[2]], d]][[2]](*Second solution is positive*)
```

Out[149]=
$$\left\{A \to -\left(\left(f\,g\,Kd\,Kdim\left(d + Kd + KA\,Kd - \sqrt{\left((d + Kd + KA\,Kd)^2 + \frac{8\left(d^2 + 2\,d\,g\,Kd + f\,g\,Kd^2\right)RAF}{f\,g\,Kdim}\right)}\right)\right)\Big/\left(4\left(d^2 + 2\,d\,g\,Kd + f\,g\,Kd^2\right)\right)\right)\right\}$$

Out[150]=
$$\left\{d \to -\frac{1}{4\,A^2}g\,Kd\left(2\,A^2 + A\,f\,Kdim + f\,Kd\,Kdim - \sqrt{\left(\frac{1}{g}\left(8\,A^2\,DTOT\,f\,Kdim + g\left(2\,A^2 + A\,f\,Kdim + f\,Kd\,Kdim\right)^2\right)\right)}\right)\right\}$$

We estimate the total activity by counting all of the drug-free protomers which occur within a partly active or fully active dimer. Substituting the above solution into the expression for Raf activity and dividing by total RAF concentration, we obtain the proportionate activity as a function of total drug and other parameters.

In[151]:=
```
RafActivity[vars_] := 2 vars[[4]] + vars[[6]]
fnActiveRAF = SimplifyPars[RafActivity[vars] / RAF /. rep4 /. sol4A];
repratios = {Kdim → RAF/RAF_rel, d → Kd d_rel};
fnActiveRAF = SimplifyPars[FullSimplify[fnActiveRAF /. repratios]]
fnActiveRAF2 = FullSimplify[
  SimplifyPars[fnActiveRAF //. {(1 + KA + d_rel) → E1c, RAF_rel → E2u f g/(f g + 2 g d_rel + d_rel^2)/8}]]
```

Out[154]=
$$\left(f\,g^2\left(f + d_{rel}\right)\left(1 + KA + d_{rel} - \sqrt{\left(\left(1 + KA + d_{rel}\right)^2 + \frac{8\left(f\,g + 2\,g\,d_{rel} + d_{rel}^2\right)RAF_{rel}}{f\,g}\right)}\right)^2\right)\Big/\left(8\left(f\,g + 2\,g\,d_{rel} + d_{rel}^2\right)^2 RAF_{rel}\right)$$

Out[155]=
$$\frac{\left(E1c - \sqrt{E1c^2 + E2u}\right)^2 g\left(f + d_{rel}\right)}{E2u\left(f\,g + 2\,g\,d_{rel} + d_{rel}^2\right)}$$

Note that the above equations represent the total active RAF protomers in proportion to the total RAF kinase as a function of unbound drug (d). In order to analytically establish parameter values which correspond to activation of the kinase, we find the first derivative of this function and search for it’s zeroes.

In[156]:=
```
RafDimers[eqns_] := (eqns[[4]] + eqns[[6]] + eqns[[7]]);
fnDimers = FullSimplify[SimplifyPars[(RafDimers[vars] / RAF /. rep4 /. sol4A) /. repratios]]
SimplifyPars[fnDimers / fnActiveRAF]
```

Out[157]=
$$\left(f\,g\left(1+KA+d_{rel}-\sqrt{(1+KA+d_{rel})^2+\frac{8\left(f\,g+2\,g\,d_{rel}+d_{rel}^2\right)RAF_{rel}}{f\,g}}\right)^2\right)\Big/\left(16\left(f\,g+2\,g\,d_{rel}+d_{rel}^2\right)RAF_{rel}\right)$$

Out[158]=
$$\frac{f\,g+2\,g\,d_{rel}+d_{rel}^2}{2\,f\,g+2\,g\,d_{rel}}$$

- **1.4.2. Baseline Signaling (drug-free)**

In[159]:=
```
baselineActiveRAF = fnActiveRAF /. d_rel → 0;
rep3b = {RAF_rel → E3 (1 + KA)^2/8};
baselineActiveRAF1 = SimplifyPars[baselineActiveRAF /. rep3b]
SimplifyPars[D[baselineActiveRAF1, E3] /. rep3b]
```

Out[161]=
$$\frac{\left(-1+\sqrt{1+E3}\right)^2}{E3}$$

Out[162]=
$$\frac{\left(-1+\sqrt{1+E3}\right)^2}{E3^2\sqrt{1+E3}}$$

The derivative of baseline signaling relative to E3 is a positive definite function. Therefore, baseline signaling is a monotonically proportionate to E3, which is in turn, monotonically proportionate to RAF concentration and monotonically inverse relationship to KA.

Hence in the base model, increasing KA decreases baseline signaling and increasing RAF_{rel} increases baseline signaling. Without drug, only autoinhibition and dimerization drive the activation, as validated in above expressions.

- **1.4.3. Conditions on parameter regions for activation in response to the drug**

In[163]:=
```
dfn4 = SimplifyPars[D[fnActiveRAF, d_rel]]
```

Out[163]=
$$\frac{1}{8\left(f\,g+2\,g\,d_{rel}+d_{rel}^2\right)^3 RAF_{rel}}\;f\,g^2\left(1+KA+d_{rel}-\sqrt{(1+KA+d_{rel})^2+\frac{8\left(f\,g+2\,g\,d_{rel}+d_{rel}^2\right)RAF_{rel}}{f\,g}}\right)\left(2\,(f+d_{rel})\left(f\,g+2\,g\,d_{rel}+d_{rel}^2\right)\left(1-\left(1+KA+d_{rel}+\frac{8\,(g+d_{rel})\,RAF_{rel}}{f\,g}\right)\Big/\left(\sqrt{(1+KA+d_{rel})^2+\frac{8\left(f\,g+2\,g\,d_{rel}+d_{rel}^2\right)RAF_{rel}}{f\,g}}\right)\right)-4\,(f+d_{rel})\,(g+d_{rel})\left(1+KA+d_{rel}-\sqrt{(1+KA+d_{rel})^2+\frac{8\left(f\,g+2\,g\,d_{rel}+d_{rel}^2\right)RAF_{rel}}{f\,g}}\right)+\left(f\,g+2\,g\,d_{rel}+d_{rel}^2\right)\left(1+KA+d_{rel}-\sqrt{(1+KA+d_{rel})^2+\frac{8\left(f\,g+2\,g\,d_{rel}+d_{rel}^2\right)RAF_{rel}}{f\,g}}\right)\right)$$

The first product term in the numerator is negative definite being of the form x – Sqrt[x^2+<positive term>]. We evaluate the conditions under which the second term may also be negative.

In[164]:= `dfn4[[-1]]`

Out[164]=
$$2\,(f + d_{rel})\,\left(f\,g + 2\,g\,d_{rel} + d_{rel}^2\right)\left(1 - \left(1 + KA + d_{rel} + \frac{8\,(g + d_{rel})\,RAF_{rel}}{f\,g}\right)\Big/\left(\sqrt{\left((1 + KA + d_{rel})^2 + \frac{8\,\left(f\,g + 2\,g\,d_{rel} + d_{rel}^2\right)RAF_{rel}}{f\,g}\right)}\right)\right) - 4\,(f + d_{rel})\,(g + d_{rel})\left(1 + KA + d_{rel} - \sqrt{\left((1 + KA + d_{rel})^2 + \frac{8\,\left(f\,g + 2\,g\,d_{rel} + d_{rel}^2\right)RAF_{rel}}{f\,g}\right)}\right) + \left(f\,g + 2\,g\,d_{rel} + d_{rel}^2\right)\left(1 + KA + d_{rel} - \sqrt{\left((1 + KA + d_{rel})^2 + \frac{8\,\left(f\,g + 2\,g\,d_{rel} + d_{rel}^2\right)RAF_{rel}}{f\,g}\right)}\right)$$

In[165]:=
```
rd4 = Reduce[dfn4[[-1]] < 0];
existPA4 = SimplifyPars[rd4]
```

Out[166]=
$$1 + KA > 2\,f + d_{rel}\ \&\&\ \Big(\Big(g == 1\ \&\&\ RAF_{rel} < \Big(f\,\left(f\,\left(4\,f^2 - 8\,f\,(1 + KA) + 3\,(1 + KA)^2\right) + \left(-8\,f^2\,KA + 2\,(1 + KA)^2 + f\,\left(-6 - 2\,KA + 4\,KA^2\right)\right)d_{rel} - \left(1 + f + 4\,f^2 - 2\,KA + 8\,f\,KA - 3\,KA^2\right)d_{rel}^2 - 2\,(2\,f + KA)\,d_{rel}^3 - d_{rel}^4\right)\Big)\Big/\left(8\,\left(f + 2\,f\,d_{rel} + d_{rel}^2\right)^2\right)\Big)\ ||\ \Big(g == 2\ \&\&\ \Big(f\,\left(-2\,f\,\left(4\,f^2 - 8\,f\,(1 + KA) + 3\,(1 + KA)^2\right) - 4\,\left(-2\,f^2\,(-1 + KA) + (1 + KA)^2 + f\,\left(-4 - 3\,KA + KA^2\right)\right)d_{rel} + \left(5 + 4\,f^2 + 2\,KA - 3\,KA^2 + f\,(-6 + 8\,KA)\right)d_{rel}^2 + 2\,(-1 + 2\,f + KA)\,d_{rel}^3 + d_{rel}^4\right)\Big)\Big/\left(4\,\left(2\,f + 2\,f\,d_{rel} + d_{rel}^2\right)^2\right) + RAF_{rel} < 0\Big)\ ||\ \Big(RAF_{rel} < \Big(f\,g\,(-1 + 2\,f - KA + d_{rel})\,\left(f\,g\,(2\,f - 3\,(1 + KA)) + (3\,f\,g - 4\,f\,(1 + KA) - 2\,g\,(1 + KA))\,d_{rel} - (3 + 2\,f - 2\,g + 3\,KA)\,d_{rel}^2 - d_{rel}^3\right)\Big)\Big/\left(8\,\left(f\,g + 2\,f\,d_{rel} + d_{rel}^2\right)^2\right)\ \&\&\ (1 < g < 2\ ||\ g < 1\ ||\ g > 2)\Big)\Big)$$

Conditions for positive derivative of active RAF relative to unbound drug are shown above.

In[167]:= `existPAU = FullSimplify[existPA4, {g >= 1, f <= 1, f > 0, KA >= 1}]`

Out[167]=
$$1 + KA > 2\,f + d_{rel}\ \&\&\ \Big(\Big(g == 1\ \&\&\ RAF_{rel} < \left(f\,(-1 + 2\,f - KA + d_{rel})\,\left(f\,(2\,f - 3\,(1 + KA)) - d_{rel}\,\left(2 + f + 2\,KA + 4\,f\,KA + d_{rel}\,(1 + 2\,f + 3\,KA + d_{rel})\right)\right)\right)\Big/\left(8\,\left(f + 2\,f\,d_{rel} + d_{rel}^2\right)^2\right)\Big)\ ||\ \Big(g == 2\ \&\&\ RAF_{rel} < \left(f\,(-1 + 2\,f - KA + d_{rel})\,\left(2\,f\,(2\,f - 3\,(1 + KA)) - d_{rel}\,\left(4 - 2\,f + 4\,KA + 4\,f\,KA + d_{rel}\,(-1 + 2\,f + 3\,KA + d_{rel})\right)\right)\right)\Big/\left(4\,\left(2\,f + 2\,f\,d_{rel} + d_{rel}^2\right)^2\right)\Big)\ ||\ \Big(RAF_{rel} < \left(f\,g\,(-1 + 2\,f - KA + d_{rel})\,\left(f\,g\,(2\,f - 3\,(1 + KA)) - d_{rel}\,\left(2\,g\,(1 + KA) + f\,(4 - 3\,g + 4\,KA) + d_{rel}\,(3 + 2\,f - 2\,g + 3\,KA + d_{rel})\right)\right)\right)\Big/\left(8\,\left(f\,g + 2\,f\,d_{rel} + d_{rel}^2\right)^2\right)\ \&\&\ (1 < g < 2\ ||\ g > 2)\Big)\Big)$$

The above condition is quite complex and dependent strongly on the drug concentration.

Also, the smallest value of d_{rel} is 0, corresponding to which a condition can be set upon f and RAF_{rel} for PA to exist.

In[168]:= `existPAU0 = SimplifyPars[existPAU /. d`$_{rel}$` → 0]`

Out[168]= $g \geq 1\ \&\&\ 8\,(f + f\,KA + RAF_{rel}) < 4\,f^2 + 3\,(1 + KA)^2\ \&\&\ 1 + KA > 2\,f$

In[169]:= `Reduce[existPAU0, RAF`$_{rel}$`]`

Out[169]= $KA \in \mathbb{R}\ \&\&\ g \geq 1\ \&\&\ f < \frac{1 + KA}{2}\ \&\&\ RAF_{rel} < \frac{1}{8}\left(3 - 8\,f + 4\,f^2 + 6\,KA - 8\,f\,KA + 3\,KA^2\right)$

In[170]:= **FullSimplify**$\left[\frac{1}{8}\left(3 - 8\,f + 4\,f^2 + 6\,KA - 8\,f\,KA + 3\,KA^2\right)\right]$

Out[170]= $\frac{1}{8}\left(4\,f^2 - 8\,f\,(1 + KA) + 3\,(1 + KA)^2\right)$

Since PA is experimentally observed at smaller values for the drug (in other words, inhibition then activation phenotype is not observed in dose response), the derivative of active RAF limiting to zero drug concentration can only be positive if above conditions are satisfied.

However, when the above conditions are satisfied, will PA always exist? Yes.

Can PA exist even when above conditions are not satisfied? That is the first derivative of active raf relative to drug is negative near zero drug but then becomes positive? And then negative again (since high drug conc have to go to zero)? - the possibility cannot be ruled out at this stage.

■ 1.4.4. Monotonic relationship between unbound (d) and total (DTOT) drug concentrations

In[171]:=
```
eqnsconsrv[[2]]
```

Out[171]=
$$d + \frac{A\,d}{Kd} + \frac{2\,A^2\,d\,(d + g\,Kd)}{f\,g\,Kd^2\,Kdim} == DTOT$$

The derivative of the function DTOT relative to d is the sum of the derivative of different terms within the second equation in expression above. Among these the derivative of the first term is 1. And the derivative of the second and third term are shown as non-negative functions of unbound drug d, below.

In[172]:=
```
sd11 = FullSimplify[SimplifyPars[D[A*d /. sol4A, d]]];
rd11 = Reduce[sd11 < 0];
SimplifyPars[rd11]
```

Out[174]= False

In[175]:=
```
sd12 = SimplifyPars[D[Simplify[2 A^2 d (d + g Kd) /. sol4A], d]]
```

Out[175]=
$$\frac{1}{8\,(d^2 + 2\,d\,g\,Kd + f\,g\,Kd^2)^3} f^2\,g^2\,Kd^2\,Kdim^2 \left(d + Kd + KA\,Kd - \sqrt{\left((d + Kd + KA\,Kd)^2 + \frac{8\,(d^2 + 2\,d\,g\,Kd + f\,g\,Kd^2)\,RAF}{f\,g\,Kdim}\right)}\right) \left(2\,d\,(d + g\,Kd)\,(d^2 + 2\,d\,g\,Kd + f\,g\,Kd^2) \left(1 - \left(d + Kd + KA\,Kd + \frac{8\,(d + g\,Kd)\,RAF}{f\,g\,Kdim}\right) \middle/ \left(\sqrt{\left((d + Kd + KA\,Kd)^2 + \frac{8\,(d^2 + 2\,d\,g\,Kd + f\,g\,Kd^2)\,RAF}{f\,g\,Kdim}\right)}\right)\right) - 4\,d\,(d + g\,Kd)^2 \left(d + Kd + KA\,Kd - \sqrt{\left((d + Kd + KA\,Kd)^2 + \frac{8\,(d^2 + 2\,d\,g\,Kd + f\,g\,Kd^2)\,RAF}{f\,g\,Kdim}\right)}\right) + d\,(d^2 + 2\,d\,g\,Kd + f\,g\,Kd^2) \left(d + Kd + KA\,Kd - \sqrt{\left((d + Kd + KA\,Kd)^2 + \frac{8\,(d^2 + 2\,d\,g\,Kd + f\,g\,Kd^2)\,RAF}{f\,g\,Kdim}\right)}\right) + (d + g\,Kd)\,(d^2 + 2\,d\,g\,Kd + f\,g\,Kd^2) \left(d + Kd + KA\,Kd - \sqrt{\left((d + Kd + KA\,Kd)^2 + \frac{8\,(d^2 + 2\,d\,g\,Kd + f\,g\,Kd^2)\,RAF}{f\,g\,Kdim}\right)}\right)\right)$$

Note that the first non-trivial product term in this expression is negative definite as the term inside the square root is always greater than the term outside. So we focus on the second term.

In[176]:=
```
sd121 = Simplify[sd12[[-1]]];
rd12 = Reduce[sd121 > 0];
SimplifyPars[rd12]
```

Out[178]=

$$\Big(\text{RAF} < \big(f\,(d\,(g-2\,(1+\text{KA})) - g\,(1+\text{KA})\,\text{Kd})\,\big(d^3\,(3\,g-2\,(1+\text{KA})) + d^2\,g\,(-3+4\,f+2\,g-3\,\text{KA})\,\text{Kd} + d\,g\,(-2\,g\,(1+\text{KA}) + f\,(2+3\,g+2\,\text{KA}))\,\text{Kd}^2 + f\,g^2\,(1+\text{KA})\,\text{Kd}^3\big)\,\text{Kdim}\big) \big/ \big(8\,g\,(d^2+2\,d\,f\,\text{Kd}+f\,g\,\text{Kd}^2)^2\big)\ \&\&\ g \neq 1\ \&\&\ 2\,g \neq 1\ \&\&\ 2\,g \neq 3\ \&\&\ \Big(\big(g\,\text{Kd}^2\,(2\,d\,(f-g)+f\,g\,\text{Kd}) > d^2\,(2\,d+3\,g\,\text{Kd})\ \&\&\ \text{KA} < \big(d^3\,(-2+3\,g) + d^2\,g\,(-3+4\,f+2\,g)\,\text{Kd} + d\,g\,(-2\,g+f\,(2+3\,g))\,\text{Kd}^2 + f\,g^2\,\text{Kd}^3\big) \big/ \big(2\,d^3+3\,d^2\,g\,\text{Kd}+2\,d\,g\,(-f+g)\,\text{Kd}^2 - f\,g^2\,\text{Kd}^3\big)\big)\ ||\ (1+\text{KA})\,(2\,d+g\,\text{Kd}) < d\,g\Big)\Big)\ ||$$

$$\left(f > \frac{2\,d\,(4\,d^2+3\,d\,\text{Kd}+\text{Kd}^2)}{\text{Kd}^2\,(4\,d+\text{Kd})}\ \&\&\ 2\,g == 1\ \&\&\ \text{RAF} < \big(f\,(3\,d+4\,d\,\text{KA}+\text{Kd}+\text{KA}\,\text{Kd})\,\big(d^3\,(2+8\,\text{KA}) + 2\,d^2\,(2-4\,f+3\,\text{KA})\,\text{Kd} + d\,(2-7\,f+2\,\text{KA}-4\,f\,\text{KA})\,\text{Kd}^2 - f\,(1+\text{KA})\,\text{Kd}^3\big)\,\text{Kdim}\big) \big/ \big(8\,(2\,d^2+4\,d\,f\,\text{Kd}+f\,\text{Kd}^2)^2\big)\ \&\&\ \text{KA} < \big(-2\,d^3+4\,d^2\,(-1+2\,f)\,\text{Kd}+d\,(-2+7\,f)\,\text{Kd}^2+f\,\text{Kd}^3\big) \big/ \big(8\,d^3+6\,d^2\,\text{Kd}+2\,d\,(1-2\,f)\,\text{Kd}^2-f\,\text{Kd}^3\big)\right)\ ||$$

$$\left(\text{Kd}^2\,(2\,d\,(-1+f)+f\,\text{Kd}) > d^2\,(2\,d+3\,\text{Kd})\ \&\&\ g == 1\ \&\&\ \text{RAF} < \big(f\,(d+2\,d\,\text{KA}+\text{Kd}+\text{KA}\,\text{Kd})\,\big(d^3\,(-1+2\,\text{KA}) + d^2\,(1-4\,f+3\,\text{KA})\,\text{Kd} + d\,(2-5\,f+2\,\text{KA}-2\,f\,\text{KA})\,\text{Kd}^2 - f\,(1+\text{KA})\,\text{Kd}^3\big)\,\text{Kdim}\big) \big/ \big(8\,(d^2+2\,d\,f\,\text{Kd}+f\,\text{Kd}^2)^2\big)\ \&\&\ \text{KA} < \frac{(d+\text{Kd})\,(d^2+2\,d\,(-1+2\,f)\,\text{Kd}+f\,\text{Kd}^2)}{2\,d^3+3\,d^2\,\text{Kd}-2\,d\,(-1+f)\,\text{Kd}^2-f\,\text{Kd}^3}\right)\ ||$$

$$\Big(3\,\text{Kd}^2\,(d\,(-6+4\,f)+3\,f\,\text{Kd}) > 2\,d^2\,(4\,d+9\,\text{Kd})\ \&\&\ 2\,g == 3\ \&\&\ \big(f\,(d+4\,d\,\text{KA}+3\,(1+\text{KA})\,\text{Kd})\,\big(d^3\,(10-8\,\text{KA}) + 6\,d^2\,(4\,f-3\,\text{KA})\,\text{Kd} + 3\,d\,(-6\,(1+\text{KA})+f\,(13+4\,\text{KA}))\,\text{Kd}^2 + 9\,f\,(1+\text{KA})\,\text{Kd}^3\big)\,\text{Kdim}\big) \big/ \big(24\,(2\,d^2+4\,d\,f\,\text{Kd}+3\,f\,\text{Kd}^2)^2\big) + \text{RAF} < 0\ \&\&\ \text{KA} < \big(10\,d^3+24\,d^2\,f\,\text{Kd}+3\,d\,(-6+13\,f)\,\text{Kd}^2+9\,f\,\text{Kd}^3\big) \big/ \big(8\,d^3+18\,d^2\,\text{Kd}-6\,d\,(-3+2\,f)\,\text{Kd}^2-9\,f\,\text{Kd}^3\big)\Big)$$

In[190]:=
```
SimplifyPars[rd12 && existPAU0]
```

Out[190]= $Aborted

rd12 needs more detailed analysis.

DTOT=d+sd11+sd12

In[179]:=
```
fnDTOT = SimplifyPars[eqnsconsrv[[2]][[1]] /. rep4 /. sol4A]
```

Out[179]=

$$\frac{1}{8}\,d\left(8 - \left(2\,f\,g\,\text{Kdim}\left(d+\text{Kd}+\text{KA}\,\text{Kd} - \sqrt{(d+\text{Kd}+\text{KA}\,\text{Kd})^2 + \frac{8\,(d^2+2\,d\,g\,\text{Kd}+f\,g\,\text{Kd}^2)\,\text{RAF}}{f\,g\,\text{Kdim}}}\right)\right) \Big/ (d^2+2\,d\,g\,\text{Kd}+f\,g\,\text{Kd}^2) + \left(f\,g\,(d+g\,\text{Kd})\,\text{Kdim}\left(d+\text{Kd}+\text{KA}\,\text{Kd} - \sqrt{(d+\text{Kd}+\text{KA}\,\text{Kd})^2 + \frac{8\,(d^2+2\,d\,g\,\text{Kd}+f\,g\,\text{Kd}^2)\,\text{RAF}}{f\,g\,\text{Kdim}}}\right)^2\right) \Big/ (d^2+2\,d\,g\,\text{Kd}+f\,g\,\text{Kd}^2)^2\right)$$

In[180]:=
```
Sty[x_] := Style[x, 20, FontFamily → "Arial"];
KTarr = {1.×10^-6, 1.×10^-7, 1.×10^-8};
kdarr = {Kd → 1.×10^-7 , Kd → 1.×10^-9};
pltfn = Flatten[Table[fnDTOT /. kd, {kd, kdarr}, {RAF, KTarr}]] /. baseparams;
ps = {Blue, Green, Directive[Red, Thick], Directive[Blue, Dashed],
   Directive[Green, Dashed], Directive[Red, Dashed, Thick]};
LogLogPlot[pltfn, {d, 10^-9, 10^-5}, Frame → True, FrameTicksStyle → 20, PlotStyle → ps,
 PlotLegends → Placed[LineLegend[ {Blue, Green, Directive[Red, Thick]}, Sty /@ KTarr,
    LegendLabel → Sty["RAF (M)"], LegendFunction → Panel, LabelStyle → 12], {Right, Bottom}],
 FrameLabel → (Sty /@ {"Unbound Drug [d] (M)", "Total Drug [DTOT] (M)"}), ImageSize → {500},
 FrameStyle → Thickness[0.004], PlotLabel → Sty["Kd: —100nM  - -1nM KA:10"]
 (*N[TableForm[parset01,TableDirections→Row]]*)]
```

Out[185]=

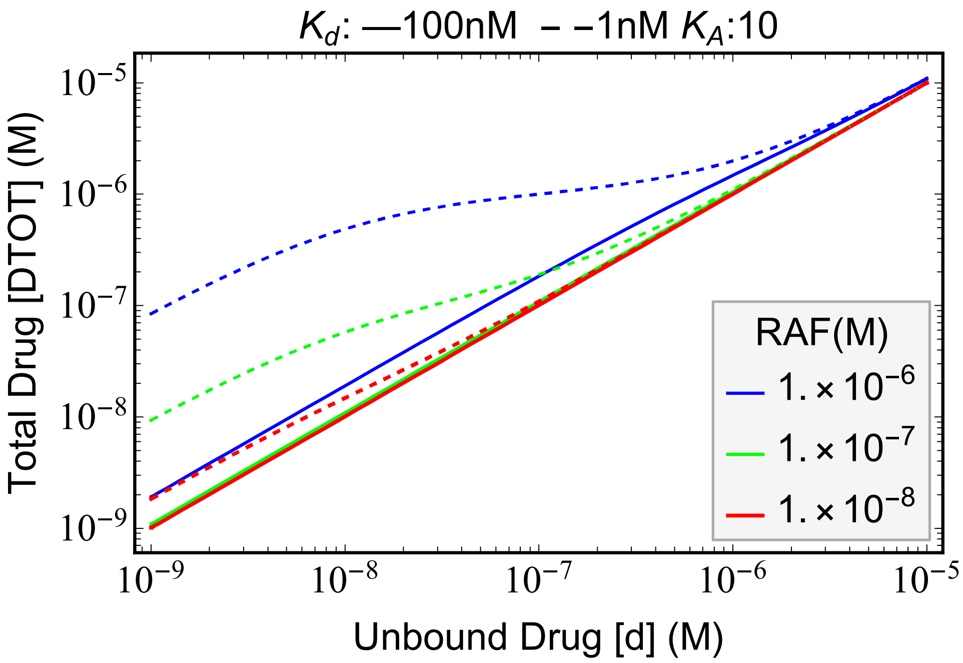

- **1.4.5. Analytic Expressions for maximum Fold Change (FC)**

Fold change is defined as the ratio between maximum RAF activity (no/conc of active protomers) to that in absence of drug

In[191]:=
```
rafFC = SimplifyPars[fnActiveRAF / baselineActiveRAF]
```

Out[191]=

$$\left(f\, g^2\, (f + d_{rel}) \left(1 + KA + d_{rel} - \sqrt{\left((1 + KA + d_{rel})^2 + \frac{8\,(f\,g + 2\,g\,d_{rel} + d_{rel}^2)\,RAF_{rel}}{f\,g}\right)}\right)^2\right) \Big/ \left((f\,g + 2\,g\,d_{rel} + d_{rel}^2)^2 \left(1 + KA - \sqrt{(1 + KA)^2 + 8\,RAF_{rel}}\right)^2\right)$$

Activating Range is defined as the lowest concentration above which the drug no longer acts as a paradoxical activator and becomes an inhibitor. This function is not easily solved analytically, and to understand its variation within the range of parameters defined in conditions for PA above, is calculated numerically.

- **1.4.6. Convert to Python**

- **Section 1.5. Unified Model: fully-analytic solution to special case where g=f**

In[ ]:=
```
Quit[]
```

- **1.5.1. Analytic Solution to the model**

This model is a solvable limit to the general all-mechanisms model and can therefore be used to test some of the analytic results. However, it is not compatible with the data. f<1 and g>1 respectively support PA mechanism and f>1 and g<1 do not. Therefore f=g with PA extant would mean that the two mechanisms act in opposite manner relative to PA. This may still be instructive as a theoretical limit applicable in dimer kinase inhibition where positive cooperativity as well as dimer stabilization mechanisms exist.

In[284]:=
```
vars = {a, A, d, AA, Ad, AAd, AdAd};(*A list of all variables*)
rep5 = {AA → A^2/Kdim, AAd → 2 A^2 d/(f Kdim Kd), AdAd → A^2 d^2/(f g Kdim Kd^2), Ad → A d/Kd, a → A KA} /. g → f;
(*derived from equilibrium relationships*)
Consrv[eqns_] :=
 {Simplify[eqns[[1]] + eqns[[2]] + eqns[[5]] + 2 (eqns[[4]] + eqns[[6]] + eqns[[7]])],
  Simplify[eqns[[3]] + eqns[[5]] + eqns[[6]] + 2 (eqns[[7]])]};
(*conservation relations for RAF and DTOT*)
eqnsconsrv = Simplify[Thread[Simplify[Consrv[vars] /. rep5] == {RAF, DTOT}]]
```

Out[285]= $\left\{A\, f\, (d + Kd + KA\, Kd) + \frac{2 A^2 (d + f\, Kd)^2}{f\, Kd\, Kdim} == f\, Kd\, RAF,\; d + \frac{A\, d}{Kd} + \frac{2 A^2 d\, (d + f\, Kd)}{f^2\, Kd^2\, Kdim} == DTOT\right\}$

A simultaneous solution to both the above conservation equations is unwieldy and hard to obtain. Instead, we find partial solutions for kinase protomers as a function of free drug and then free drug as a function of the total drug and the kinase protomers. The latter solution is used to numerically construct d vs DTOT relationship.

In[286]:=
```
SimplifyPars[x_] :=
  Simplify[x, {RAF > 0, DTOT > 0, Kd > 0, Kdim > 0, KA > 0, d > 0, f > 0, g > 0, d_rel > 0, RAF_rel > 0}];
sol5A = SimplifyPars[Solve[eqnsconsrv[[1]], A]][[2]] (*Second solution is positive*)
sol5d = SimplifyPars[Solve[eqnsconsrv[[2]], d]][[2]](*Second solution is positive*)
```

Out[287]= $\left\{A \to -\left(\left(f\, Kd\, Kdim\left(d\, f + f\, Kd + f\, KA\, Kd - \sqrt{\left(f^2 (d + Kd + KA\, Kd)^2 + \frac{8 (d + f\, Kd)^2 RAF}{Kdim}\right)}\right)\right) \Big/ \left(4 (d + f\, Kd)^2\right)\right)\right\}$

Out[288]= $\left\{d \to -\frac{1}{4 A^2} f\, Kd \left(2 A^2 + A\, f\, Kdim + f\, Kd\, Kdim - \sqrt{\left(8 A^2\, DTOT\, Kdim + \left(2 A^2 + A\, f\, Kdim + f\, Kd\, Kdim\right)^2\right)}\right)\right\}$

We estimate the total activity by counting all of the drug-free protomers which occur within a partly active or fully active dimer. Substituting the above solution into the expression for Raf activity and dividing by total RAF concentration, we obtain the proportionate activity as a function of total drug and other parameters.

In[289]:=
```
RafActivity[vars_] := 2 vars[[4]] + vars[[6]]
fnActiveRAF5 = SimplifyPars[RafActivity[vars] / RAF /. rep5 /. sol5A];
repratios = {Kdim → RAF/RAF_rel, d → Kd d_rel};
fnActiveRAF5 = SimplifyPars[FullSimplify[fnActiveRAF5 /. repratios]]
fnActiveRAF52 = FullSimplify[
  SimplifyPars[fnActiveRAF5 //. {KA → E1c - 1 - d_rel, RAF_rel → E2u2 * f / (f + d_rel (2 + f d_rel)) / 8}]]
```

Out[292]= $\left(f\left(f + f\, KA + f\, d_{rel} - \sqrt{\left(f^2 (1 + KA + d_{rel})^2 + 8 (f + d_{rel})^2 RAF_{rel}\right)}\right)^2\right) \Big/ \left(8 (f + d_{rel})^3 RAF_{rel}\right)$

Out[293]= $\left((f + d_{rel} (2 + f\, d_{rel}))\left(-E1c\, f + \sqrt{f\left(E1c^2 f + \frac{E2u2 (f + d_{rel})^2}{f + d_{rel} (2 + f\, d_{rel})}\right)}\right)^2\right) \Big/ \left(E2u2 (f + d_{rel})^3\right)$

Note that the above equations represent the total active RAF protomers in proportion to the total RAF kinase as a function of unbound drug (d). In order to analytically establish parameter values which correspond to activation of the kinase, we find the first derivative of this function (equation 11) and search for it's zeroes (equation 12).

In[294]:=
```
RafDimers[eqns_] := (eqns[[4]] + eqns[[6]] + eqns[[7]]);
fnDimers5 = FullSimplify[SimplifyPars[(RafDimers[vars] / RAF /. rep5 /. sol5A) /. repratios]]
SimplifyPars[fnDimers5 / fnActiveRAF5]
```

Out[295]=
$$\left(-f\,(1+KA+d_{rel})+\sqrt{f^2\,(1+KA+d_{rel})^2+8\,(f+d_{rel})^2\,RAF_{rel}}\right)^2 \Big/ \left(16\,(f+d_{rel})^2\,RAF_{rel}\right)$$

Out[296]=
$$\frac{f+d_{rel}}{2\,f}$$

▫ ***4.2. Baseline Signaling (drug - free)***

In[297]:=
```
baselineActiveRAF5 = fnActiveRAF5 /. d_rel → 0;
rep3b5 = {RAF_rel → E3 (1 + KA)^2/8};
baselineActiveRAF15 = SimplifyPars[baselineActiveRAF5 /. rep3b5]
SimplifyPars[D[baselineActiveRAF15, E3] /. rep3b5]
```

Out[299]=
$$\frac{\left(-1+\sqrt{1+E3}\right)^2}{E3}$$

Out[300]=
$$\frac{\left(-1+\sqrt{1+E3}\right)^2}{E3^2\,\sqrt{1+E3}}$$

The derivative of baseline signaling relative to E3 is a positive definite function. Therefore, baseline signaling is a monotonically proportionate to E3, which is in turn, monotonically proportionate to RAF concentration and monotonically inverse relationship to KA.

Hence in the base model, increasing KA decreases baseline signaling and increasing RAF_{rel} increases baseline signaling.

▪ 1.5.3. Conditions on parameter regions for activation in response to the drug

In[301]:=
```
dfn5 = SimplifyPars[D[fnActiveRAF5, d_rel]]
```

Out[301]=
$$\frac{1}{8\,(f+d_{rel})^4\,RAF_{rel}} f \left(f+f\,KA+f\,d_{rel}-\sqrt{f^2\,(1+KA+d_{rel})^2+8\,(f+d_{rel})^2\,RAF_{rel}}\right) \left(2\,(f+d_{rel})\left(f-\frac{2\,f^2\,(1+KA+d_{rel})+16\,(f+d_{rel})\,RAF_{rel}}{2\sqrt{f^2\,(1+KA+d_{rel})^2+8\,(f+d_{rel})^2\,RAF_{rel}}}\right)-3\left(f+f\,KA+f\,d_{rel}-\sqrt{f^2\,(1+KA+d_{rel})^2+8\,(f+d_{rel})^2\,RAF_{rel}}\right)\right)$$

In[302]:=
```
zeroes5 = SimplifyPars[Solve[dfn5 == 0, d_rel, VerifySolutions → True]]
```

Out[302]=
$$\left\{\left\{d_{rel}\to-\frac{f\left(f+f\,KA+8\,RAF_{rel}+2\,\text{Abs}[1-f+KA]\sqrt{f^2+6\,RAF_{rel}}\right)}{f^2+8\,RAF_{rel}}\right\},\left\{d_{rel}\to-\frac{f\left(f+f\,KA+8\,RAF_{rel}-2\,\text{Abs}[1-f+KA]\sqrt{f^2+6\,RAF_{rel}}\right)}{f^2+8\,RAF_{rel}}\right\}\right\}$$

The first solution is negative definite. Below, we derive the rules (expression 13) which allow the second solution to be positive

In[303]:=
```
exist5 = SimplifyPars[Reduce[((d_rel /. zeroes5[[2]]) > 0) && (KA > 0) && (RAF_rel > 0)]]
```

Out[303]=
$$4\,f^2+3\,(1+KA)^2>8\,(f+f\,KA+RAF_{rel})\ \&\&\ (2\,f<1+KA\ ||\ 2\,f>3+3\,KA)$$

In[304]:= d2fn5 = SimplifyPars[D[dfn5, d_{rel}]]

Out[304]=
$$\frac{1}{8\,(f+d_{rel})^5\,RAF_{rel}}\,f\left((f+d_{rel})\left(-f-\frac{16\,f^2\,(1-f+KA)^2\,(f+d_{rel})\,RAF_{rel}}{\left(f^2\,(1+KA+d_{rel})^2+8\,(f+d_{rel})^2\,RAF_{rel}\right)^{3/2}}+\frac{2\,f^2\,(1+KA+d_{rel})+16\,(f+d_{rel})\,RAF_{rel}}{2\sqrt{f^2\,(1+KA+d_{rel})^2+8\,(f+d_{rel})^2\,RAF_{rel}}}\right)\left(f+f\,KA+f\,d_{rel}-\sqrt{f^2\,(1+KA+d_{rel})^2+8\,(f+d_{rel})^2\,RAF_{rel}}\right)+(f+d_{rel})\left(f-\frac{2\,f^2\,(1+KA+d_{rel})+16\,(f+d_{rel})\,RAF_{rel}}{2\sqrt{f^2\,(1+KA+d_{rel})^2+8\,(f+d_{rel})^2\,RAF_{rel}}}\right)\left(2\,(f+d_{rel})\left(f-\frac{2\,f^2\,(1+KA+d_{rel})+16\,(f+d_{rel})\,RAF_{rel}}{2\sqrt{f^2\,(1+KA+d_{rel})^2+8\,(f+d_{rel})^2\,RAF_{rel}}}\right)-3\left(f+f\,KA+f\,d_{rel}-\sqrt{f^2\,(1+KA+d_{rel})^2+8\,(f+d_{rel})^2\,RAF_{rel}}\right)\right)-4\left(f+f\,KA+f\,d_{rel}-\sqrt{f^2\,(1+KA+d_{rel})^2+8\,(f+d_{rel})^2\,RAF_{rel}}\right)\left(2\,(f+d_{rel})\left(f-\frac{2\,f^2\,(1+KA+d_{rel})+16\,(f+d_{rel})\,RAF_{rel}}{2\sqrt{f^2\,(1+KA+d_{rel})^2+8\,(f+d_{rel})^2\,RAF_{rel}}}\right)-3\left(f+f\,KA+f\,d_{rel}-\sqrt{f^2\,(1+KA+d_{rel})^2+8\,(f+d_{rel})^2\,RAF_{rel}}\right)\right)\right)$$

In[305]:= d2fn5z = SimplifyPars[d2fn5 /. zeroes5[[2]]]

Out[305]=
$$\begin{cases} -\left(\left(\left(f^2+8\,RAF_{rel}\right)^2\left(4\,RAF_{rel}+f\left(f-\sqrt{f^2+6\,RAF_{rel}}\right)\right)\left(RAF_{rel}\left(6\,f-4\sqrt{f^2+6\,RAF_{rel}}\right)+f^2\left(f-\sqrt{f^2+6\,RAF_{rel}}\right)\right)\right)\Big/\left(4\,f^2\,(-1+f-KA)^3\,RAF_{rel}\left(f-2\sqrt{f^2+6\,RAF_{rel}}\right)^4\right)\right) & f\le 1+KA \\ \left(\left(f^2+8\,RAF_{rel}\right)^2\left(12\,RAF_{rel}+f\left(f-\sqrt{f^2+6\,RAF_{rel}}\right)\right)\left(1296\,RAF_{rel}^2+6\,f\,RAF_{rel}\left(45\,f-16\sqrt{f^2+6\,RAF_{rel}}\right)+13\,f^3\left(f-\sqrt{f^2+6\,RAF_{rel}}\right)\right)\right)\Big/\left(4\,f^2\,(-1+f-KA)^3\,RAF_{rel}\left(f+2\sqrt{f^2+6\,RAF_{rel}}\right)^5\right) & True \end{cases}$$

In[306]:= d2fn5z[[1]][[1, 1]]

Out[306]=
$$-\left(\left(\left(f^2+8\,RAF_{rel}\right)^2\left(4\,RAF_{rel}+f\left(f-\sqrt{f^2+6\,RAF_{rel}}\right)\right)\left(RAF_{rel}\left(6\,f-4\sqrt{f^2+6\,RAF_{rel}}\right)+f^2\left(f-\sqrt{f^2+6\,RAF_{rel}}\right)\right)\right)\Big/\left(4\,f^2\,(-1+f-KA)^3\,RAF_{rel}\left(f-2\sqrt{f^2+6\,RAF_{rel}}\right)^4\right)\right)$$

In[307]:=
```
exist5d2 = SimplifyPars[SimplifyPars[Reduce[(d2fn5z[[1]][[1, 1]] < 0) && (KA >= 0) && (RAF_rel >= 0)]]]
```

Out[307]= $f < 1 \,||\, 1 + KA > f$

The f<1 condition comes naturally from the equations. The alternative is that KA>1+f so PA exists even when f>1.

In[308]:=
```
SimplifyPars[SimplifyPars[Reduce[(d2fn5z[[2]] < 0) && (KA >= 0) && (RAF_rel >= 0)]]]
```

Out[308]= $f < 1 \,||\, 1 + KA > f$

In[309]:=
```
exist5A = FullSimplify[exist5 && exist5d2]
```

Out[309]= $4 f^2 + 3 (1 + KA)^2 > 8 (f + f\,KA + RAF_{rel}) \,\&\&\, (2 f < 1 + KA \,||\, 2 f > 3 + 3 KA) \,\&\&\, (f < 1 \,||\, 1 + KA > f)$

These are the full set of conditions for PA to exist in the model. The conditions above require f<(1+KA)/2. This is because considering the last two 'AND' conditions, if f>3/2(1+KA) it cannot simultaneously satisfy either of the last two conditions. Therefore, f <(1+KA)/2 needs to be satisfied. Under this condition, if f<1, it is automatically satisfied that f<(1+KA)/2 as long as KA>1. If f<(1+KA)/2, the last condition is automatically satisfied that f<1+KA.

In[310]:=
```
exist5A1 = f < (1 + KA) / 2
```

Out[310]= $f < \frac{1 + KA}{2}$

In[311]:=
```
exist5full = SimplifyPars[Reduce[exist5A && exist5A1]]
```

Out[311]= $2 f < 1 + KA \,\&\&\, 8 (f + f\,KA + RAF_{rel}) < 4 f^2 + 3 (1 + KA)^2$

- **1.5.4. Monotonic relationship between unbound (d) and total (DTOT) drug concentrations**

The derivative of the function DTOT relative to d is the sum of the derivative of different terms within the second equation in expression (1). Among these the derivative of the first term is 1. And the derivative of the second and third term are shown as non-negative functions of unbound drug d, below.

In[312]:=
```
eqnsconsrv[[2]]
```

Out[312]= $d + \frac{A\,d}{Kd} + \frac{2 A^2 d (d + f\,Kd)}{f^2 Kd^2 Kdim} == DTOT$

In[313]:=
```
sd511 = FullSimplify[SimplifyPars[D[A*d /. sol5A, d]]];
rd511 = Reduce[sd511 < 0];
rd511s = SimplifyPars[rd511]
```

Out[315]= $f > 1 + KA \,\&\&\, \left(d (1 - 2 f + KA) == f (1 + KA) Kd \,||\, \left(RAF > \frac{(1 + KA) (d (1 - 2 f + KA) - f (1 + KA) Kd) Kdim}{8 (d + f\,Kd)} \,\&\&\, f (2 d + Kd + KA\,Kd) < d (1 + KA)\right)\right)$

Note that the first condition, f be larger than 1+KA and the conditions derived for PA, that f<(1+KA)/2 cannot be simultaneously satisfied. Therefore, drugs that display PA have positive or 0 value for the derivative sd511.

In[316]:=
```
SimplifyPars[Reduce[rd511s && exist5A1]]
```

Out[316]= False

Therefore, when PA exists, the function sd511 cannot be negative.

In[317]:=
```
sd512 = SimplifyPars[D[Simplify[A^2 d (d + f Kd) /. sol5A], d]]
```

Out[317]=

$$\frac{1}{16\,(d+f\,Kd)^4} f^2\,Kd^2\,Kdim^2 \left(d\,f + f\,Kd + f\,KA\,Kd - \sqrt{f^2\,(d + Kd + KA\,Kd)^2 + \frac{8\,(d+f\,Kd)^2\,RAF}{Kdim}}\right)$$
$$\left(2\,d\,(d+f\,Kd)\left(f - \frac{2\,f^2\,(d+Kd+KA\,Kd) + \frac{16\,(d+f\,Kd)\,RAF}{Kdim}}{2\sqrt{f^2\,(d+Kd+KA\,Kd)^2 + \frac{8\,(d+f\,Kd)^2\,RAF}{Kdim}}}\right) -\right.$$
$$3\,d\left(d\,f + f\,Kd + f\,KA\,Kd - \sqrt{f^2\,(d + Kd + KA\,Kd)^2 + \frac{8\,(d+f\,Kd)^2\,RAF}{Kdim}}\right) +$$
$$\left.(d+f\,Kd)\left(d\,f + f\,Kd + f\,KA\,Kd - \sqrt{f^2\,(d + Kd + KA\,Kd)^2 + \frac{8\,(d+f\,Kd)^2\,RAF}{Kdim}}\right)\right)$$

Note that the first non-trivial product term in this expression is negative definite as the term inside the square root is always greater than the term outside. So we focus on the second term.

In[318]:=
```
sd5121 = SimplifyPars[sd512[[-1]]];
rd512 = Reduce[sd5121 > 0];
```

In[320]:=
```
rd512s = SimplifyPars[SimplifyPars[rd512]]
```

Out[320]=

$$RAF < \frac{1}{8\,(d+f\,Kd)^2}\left(d^2\left(3\,f^2 - 8\,f\,(1+KA) + 4\,(1+KA)^2\right) - 2\,d\,f^2\,(1+KA)\,Kd - f^2\,(1+KA)^2\,Kd^2\right) Kdim \;\&\&$$
$$(1+KA)\,(2\,d + f\,Kd) < d\,f$$

Again, notice the second necessary condition.

In[321]:=
```
SimplifyPars[Reduce[rd512s[[-1]] && exist5A1]]
```

Out[321]= False

When PA exists, the function sd5121 is never positive. And since its coefficient is always negative, therefore sd512 is positive or 0.

Finally,

DTOT=d+sd511/Kd+sd512/(f^2 Kd^2 Kdim)

Since all three expressions on RHS are non-negative derivatives as a function of unbound drug - the total drug has a non-negative derivative. Therefore, DTOT(d) is monotonically positive/increasing function.

In[322]:=
```
fnDTOT = SimplifyPars[Consrv[vars][[2]] /. rep5 /. sol5A];
```

- **1.5.5. Analytic Expressions for maximum Fold Change (FC)**

Fold change is defined as the ratio between maximum RAF activity (no/conc of active protomers) to that in absence of drug

In[323]:=
```
maxActiveRAF5 = FullSimplify[SimplifyPars[fnActiveRAF5 /. zeroes5[[2]]]];
rafFC5 = SimplifyPars[maxActiveRAF5 / baselineActiveRAF5]
```

Out[324]=

$$\left(4\left(f^2+8\,RAF_{rel}\right)\left(4\,(-1+f-KA)\,RAF_{rel}+Abs[1-f+KA]\left(8\,RAF_{rel}+f\left(f-\sqrt{f^2+6\,RAF_{rel}}\right)\right)\right)^2\right)\Big/\left(\left(f\,(-1+f-KA)+2\,Abs[1-f+KA]\sqrt{f^2+6\,RAF_{rel}}\right)^3\left(1+KA-\sqrt{(1+KA)^2+8\,RAF_{rel}}\right)^2\right)$$

When the ‘exist’ conditions derived above to qualify for existence of PA within this model are satisfied, we can evaluate the functional dependence of the raf fold change expression on RAF_{rel} and KA:

In[325]:=
```
dRAFrafFC5 = SimplifyPars[D[rafFC5, RAF_rel]];
dKArafFC5 = SimplifyPars[D[rafFC5, KA]];
dfrafFC5 = SimplifyPars[D[rafFC5, f]];
```

In[328]:=
```
Simplify[SimplifyPars[Reduce[dRAFrafFC5 < 0]], exist5A1]
```

Out[328]= $4\,f^2+3\,(1+KA)^2>8\,(f+f\,KA+RAF_{rel})$

Under typical conditions for PA, f<1 and KA>1. Therefore the above condition is pretty easy to satisfy with RAF<<Kdim. That is when RAF has a low concentration or weakly dimerizes. In that case, RAF increase reduces fold change.
However, with stronger dimerization or very high, likely super-physical RAF concentration(~100 micro-molar), it may be possible to invert the curve, however, the same condition is derived for PA, therefore, the increasing RAF concentration will keep reducing fold change until no PA is observed. Monotonically.

In[329]:=
```
exist5full
```

Out[329]= $2\,f<1+KA\;\&\&\;8\,(f+f\,KA+RAF_{rel})<4\,f^2+3\,(1+KA)^2$

Activating Range is defined as the lowest concentration above which the drug no longer acts as a paradoxical activator and becomes an inhibitor. This function is not easily solved analytically, and to understand its variation within the range of parameters defined in conditions for PA above, is calculated numerically.

Descriptive, example Plots (supp text section 3)

Plots for individual curves. Run all 1st sub sections (*.1) for any ONE of sections 1-4 to get the following plots.

In[ ]:=
```
Sty[x_] := Style[x, 22, Bold, FontFamily → "Times"];
```

In[ ]:=
```
plfn1 = ({fnActiveRAFDS / (fnActiveRAFDS /. d_rel → 0),
      fnActiveRAFNC / (fnActiveRAFNC /. d_rel → 0), fnActiveRAFCA / (fnActiveRAFCA /. d_rel → 0),
      fnActiveRAF / (fnActiveRAF /. d_rel → 0)} /. {RAF_rel → RAF / Kdim} /. baseparams);
```

In[ ]:=
```
legendlist = Sty["AK\n"];
rvals = {2., 0.03, 0.07, 0.02};
r1 = {Tiny, Small, Medium, Large};
cols = {Blue, Orange, Green, Black};
cs = Table[Directive[ Thickness[0.01], cols[[i]], Dashing[{rvals[[i]], r1[[i]]}]], {i, 4}];
(*dashing style by model*)
LogPlot[plfn1, {drel, 0, 100}, Frame -> True,
 (*FrameLabel→(Sty/@{"Relative Unbound Drug Concentration","RAF Complex Molecules"}),*)
 ImageSize -> {500}, FrameStyle -> Thickness[0.004], FrameTicksStyle -> Directive[25, Black],
 PlotRange -> Full, PlotStyle → cs, PlotLegends -> "AK"]
```

Out[ ]=

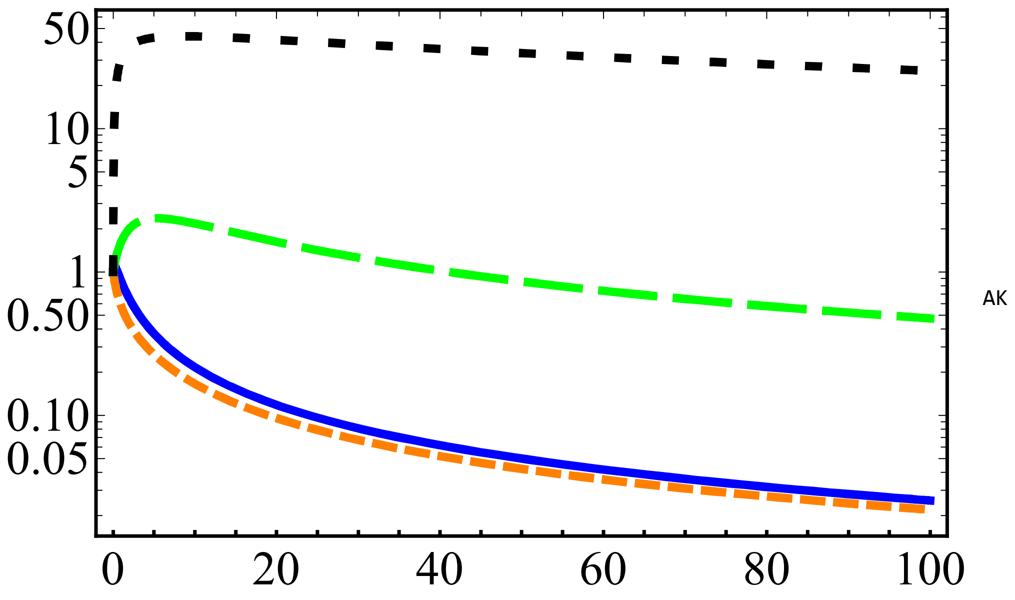

In[ ]:=
```
fn2part[1] =
  SimplifyPars[(({a, AA, AAd, AdAd, A, Ad} /. rep1 /. sol1A) / RAF /. d → (drel Kd)) /. baseparams];
fn2part[2] = SimplifyPars[
   (({a, AA, AAd, AdAd, A, Ad} /. rep2 /. sol2A) / RAF /. d → (drel Kd)) /. baseparams];
fn2part[3] = SimplifyPars[(({a, AA, AAd, AdAd, A, Ad} /. rep3 /. sol3A) / RAF /. d → (drel Kd)) /.
    baseparams];
fn2part[4] = SimplifyPars[(({a, AA, AAd, AdAd, A, Ad} /. rep4 /. sol4A) / RAF /. d → (drel Kd)) /.
    baseparams];
plfn = SimplifyPars[Table[fn2part[i][[j]], {j, 6}, {i, 4}]];
legendlist = Sty /@ { "a\n", "AA\n", " AAd\n", " AdAd\n", " A\n", " Ad\n"};
Table[Plot[Evaluate[plfn[[i]]], {drel, 0, 100}, Frame -> True, ImageSize -> {500},
  FrameStyle -> Thickness[0.004], FrameTicksStyle -> Directive[25, Black],
  PlotRange -> All, PlotStyle → cs, PlotLegends -> legendlist[[i]]], {i, Length[plfn]}]
```

Out[ ]= {

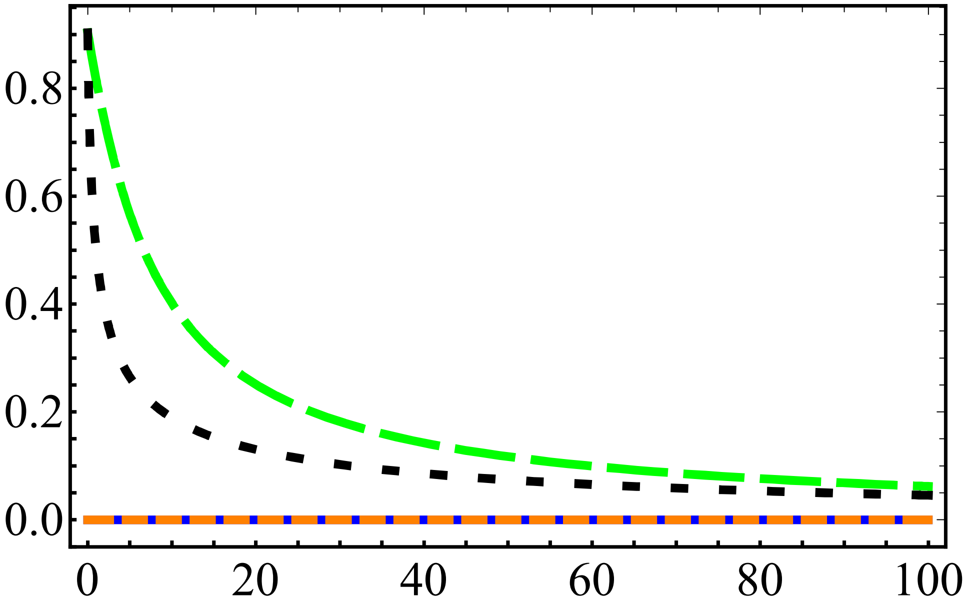

a ,

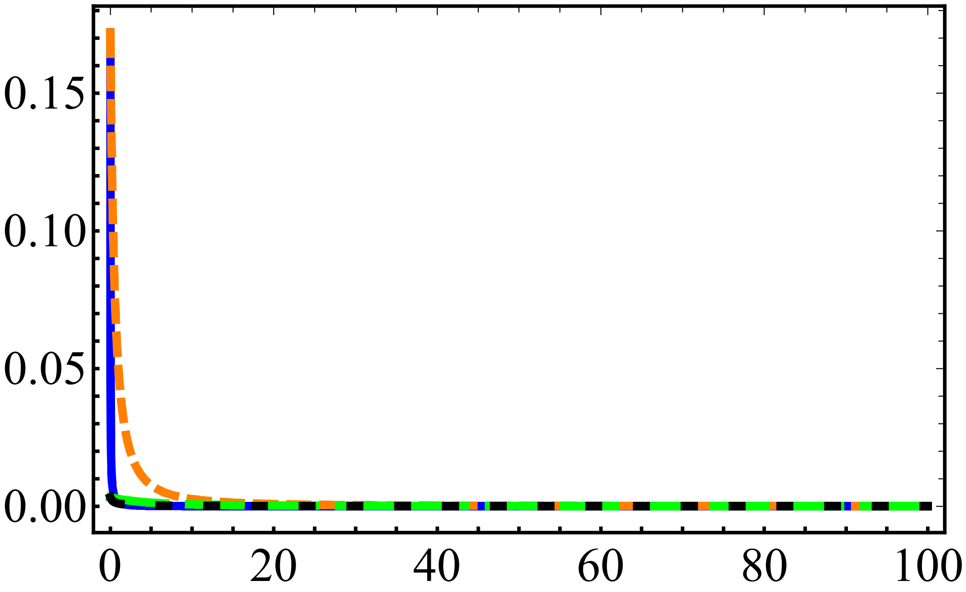

AA ,

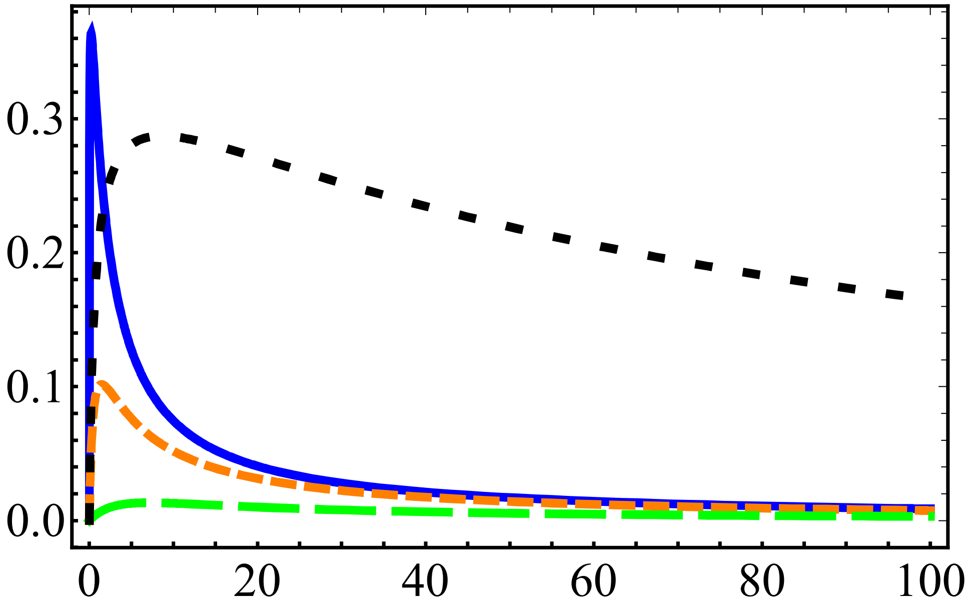

AAd ,

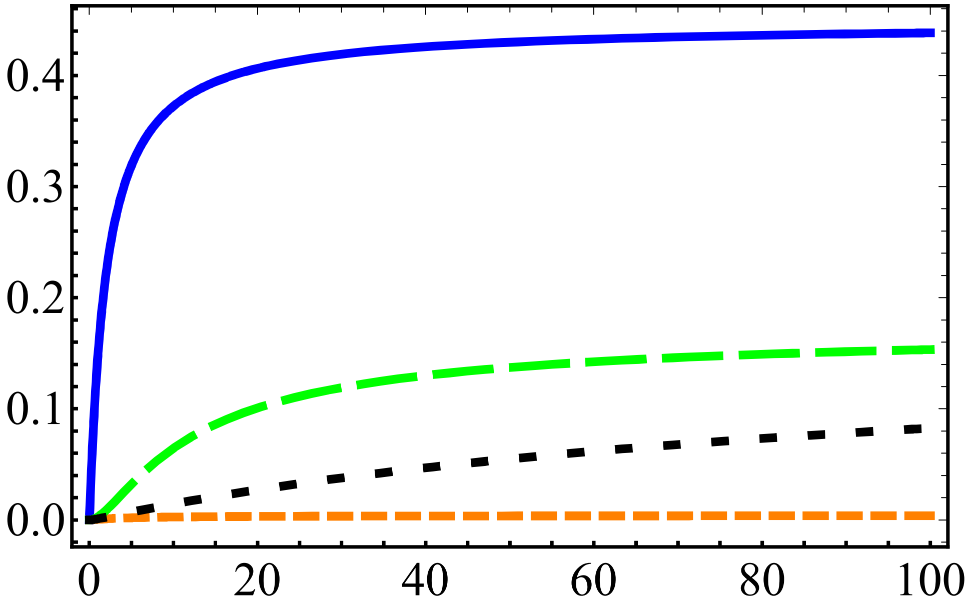

AdAd ,

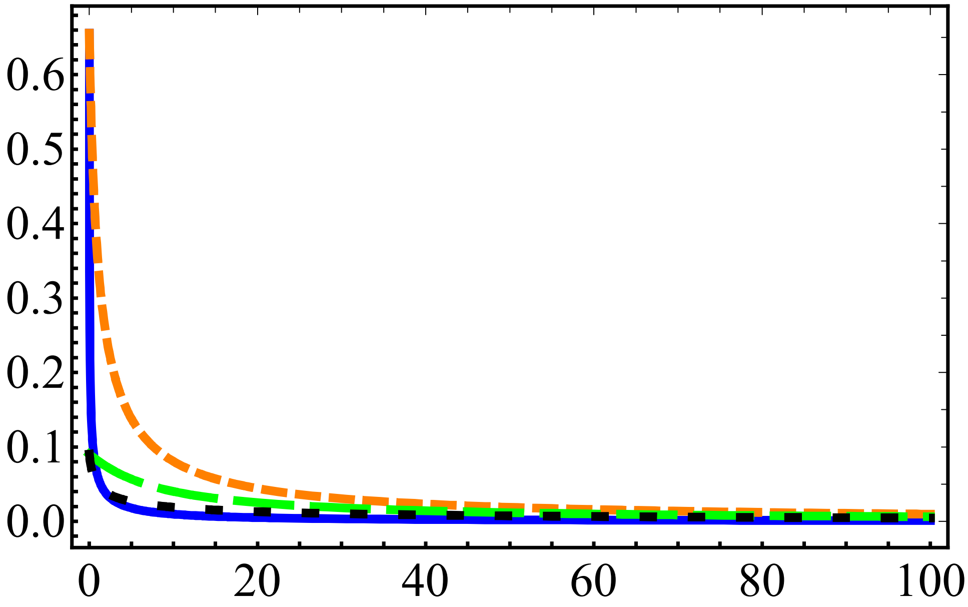
0.6
0.5
0.4
0.3
0.2
0.1
0.0
0
20
40
60
80
100

A ,

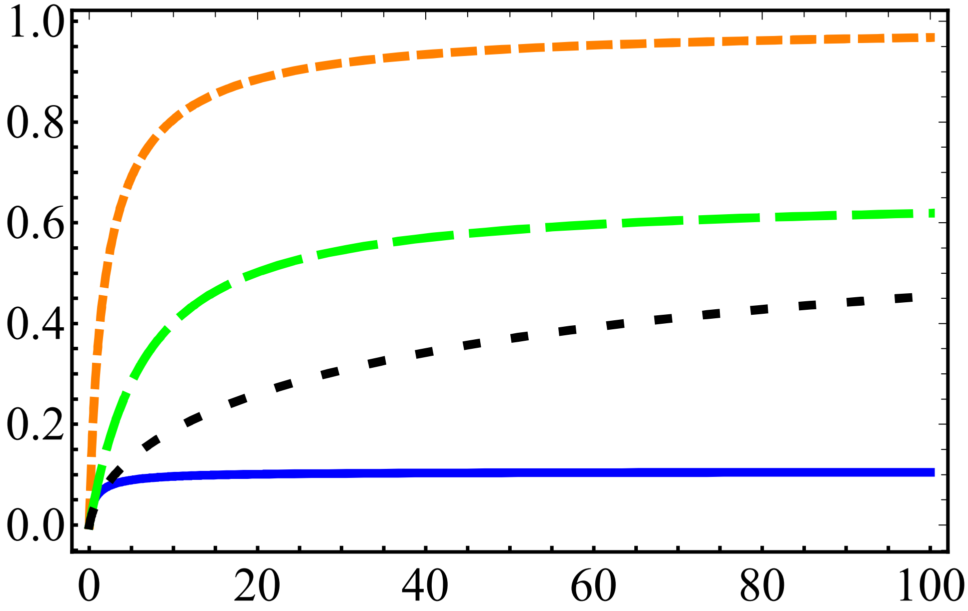
1.0
0.8
0.6
0.4
0.2
0.0
0
20
40
60
80
100

Ad}
