# Supplementary material for: Theoretical analysis reveals a role for RAF conformational autoinhibition in paradoxical activation
Source: eLife. 2023 Oct 12;12:e82739. doi: 10.7554/eLife.82739 (PMC10627510; doi:10.7554/eLife.82739)
Supplement: Source code 1. — Contents include a folder that includes Mathematica notebooks that derive the analytic results. Contents also include a folder that includes Python notebooks that reproduce all of the numerical results. [file elife-82739-code1.zip › Supplementary_code/Analytical_Validations_Mathematica_All_Models/Mathematica_AllModels_Index.pdf]

```
$Version (*Mathematica version used to generate this file on April 14 2023.*)
```

Out[78]= 12.0.0 for Microsoft Windows (64-bit) (April 6, 2019)

# 1. Distinct Mechanisms of PA

```
In[70]:= baseparams = {KA → 10, Kdim → 0.1, RAF → 0.04, f → 0.01, g → 100};
(*units: μM ∀ params but KA*)
```

## Section 1.1. Dimer Potentiation (DP) Model

```
In[ ]:= Quit[]
```

- **1.1.1. Analytic Solution to the model**
- **1.1.2. Baseline Signaling (drug-free)**
- **1.1.3. Conditions on parameter regions for activation in response to the drug**
- **1.1.4. Monotonic relationship between unbound (d) and total (DTOT) drug concentrations**
- **1.1.5. Analytic Expressions for maximum Fold Change (FC)**

## Section 1.2. Negative Cooperativity (NC) Model

```
In[ ]:= Quit[]
```

- **1.2.1. Analytic Solution to the model**
- **1.2.2. Baseline Signaling (drug-free)**
- **1.2.3. Conditions on parameter regions for activation in response to the drug**
- **1.2.5. Analytic Expressions for maximum Fold Change (FC)**

## Section 1.3. Conformal Autoinhibition (CA/base) Model

```
In[ ]:= Quit[]
```

- **1.3.1. Analytic Solution to the model**
- **1.3.2. Baseline Signaling (drug-free)**
- **1.3.3. Conditions on parameter regions for activation in response to the drug**
- **1.3.4. Monotonic relationship between unbound (d) and total (DTOT) drug concentrations**
- **1.3.5. Analytic Expressions for maximum Fold Change (FC)**

## Section 1.4. Unified Model

```
In[ ]:= Quit[]
```

- **1.4.1. Analytic Solution to the model**
- **1.4.2. Baseline Signaling (drug-free)**
- **1.4.3. Conditions on parameter regions for activation in response to the drug**

- **1.4.4. Monotonic relationship between unbound (d) and total (DTOT) drug concentrations**
- **1.4.5. Analytic Expressions for maximum Fold Change (FC)**
- **1.4.6. Convert to Python**
- **Section 1.5. Unified Model: fully-analytic solution to special case where f=g**

In[ ]:=
```
Quit[]
```

- **1.5.1. Analytic Solution to the model**
- **1.5.3. Conditions on parameter regions for activation in response to the drug**
- **1.5.4. Monotonic relationship between unbound (d) and total (DTOT) drug concentrations**
- **1.5.5. Analytic Expressions for maximum Fold Change (FC)**

# Descriptive, example Plots (supp text section 3)
