# Supplementary figures and images for: Theoretical analysis reveals a role for RAF conformational autoinhibition in paradoxical activation
Source: eLife. 2023 Oct 12;12:e82739. doi: 10.7554/eLife.82739 (PMC10627510; doi:10.7554/eLife.82739)

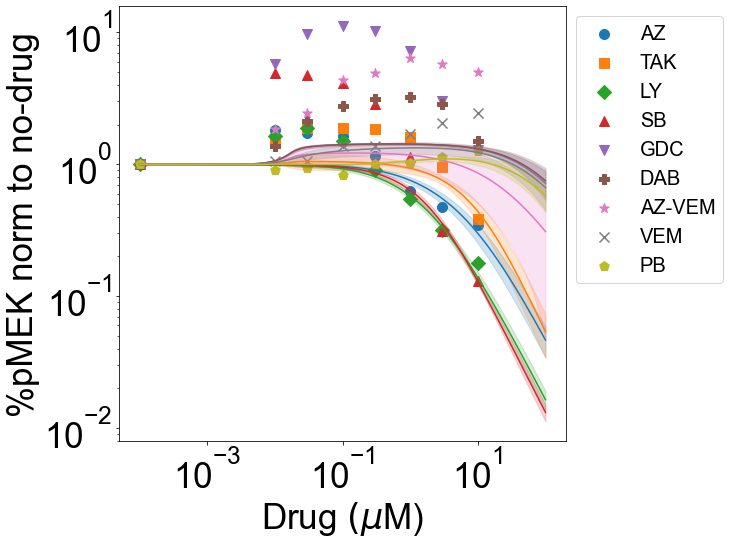

Supplement: Source code 1. — Contents include a folder that includes Mathematica notebooks that derive the analytic results. Contents also include a folder that includes Python notebooks that reproduce all of the numerical results. [file elife-82739-code1.zip › Supplementary_code/RAF_PA_Mechanisms/subModel_plots/28pars_DoseResponse.png]

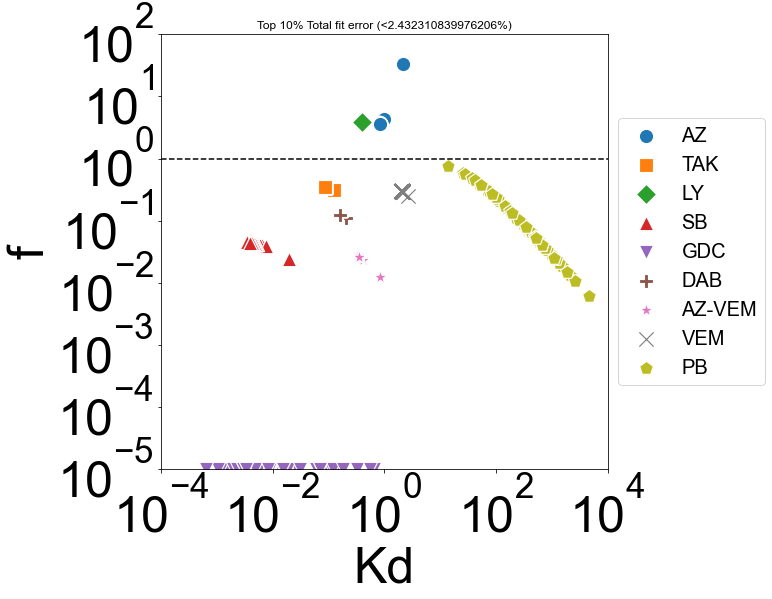

Supplement: Source code 1. — Contents include a folder that includes Mathematica notebooks that derive the analytic results. Contents also include a folder that includes Python notebooks that reproduce all of the numerical results. [file elife-82739-code1.zip › Supplementary_code/RAF_PA_Mechanisms/subModel_plots/28pars_Kd_vs_f.png]

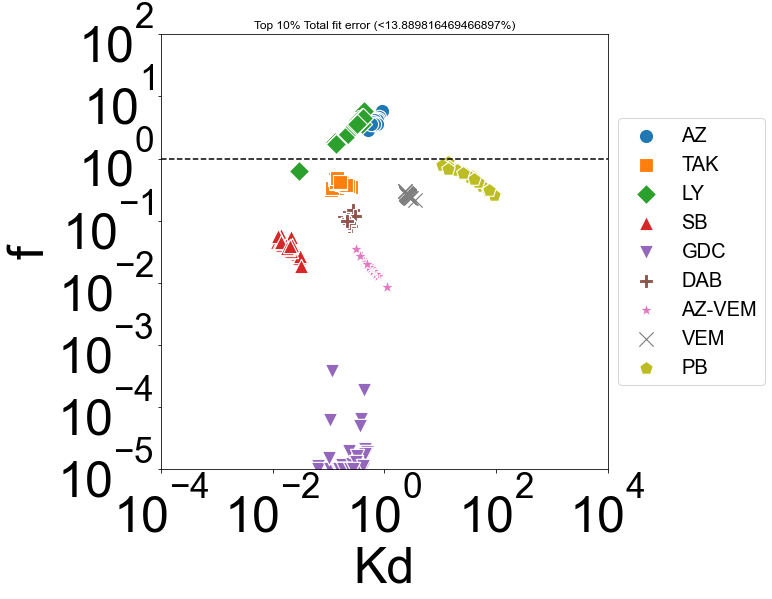

Supplement: Source code 1. — Contents include a folder that includes Mathematica notebooks that derive the analytic results. Contents also include a folder that includes Python notebooks that reproduce all of the numerical results. [file elife-82739-code1.zip › Supplementary_code/RAF_PA_Mechanisms/subModel_plots/28pars_normtp1_Kd_vs_f.png]

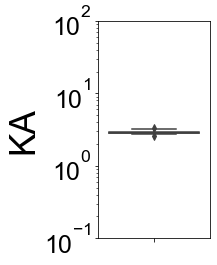

Supplement: Source code 1. — Contents include a folder that includes Mathematica notebooks that derive the analytic results. Contents also include a folder that includes Python notebooks that reproduce all of the numerical results. [file elife-82739-code1.zip › Supplementary_code/RAF_PA_Mechanisms/subModel_plots/28pars_normtp1_parameter_KA.png]

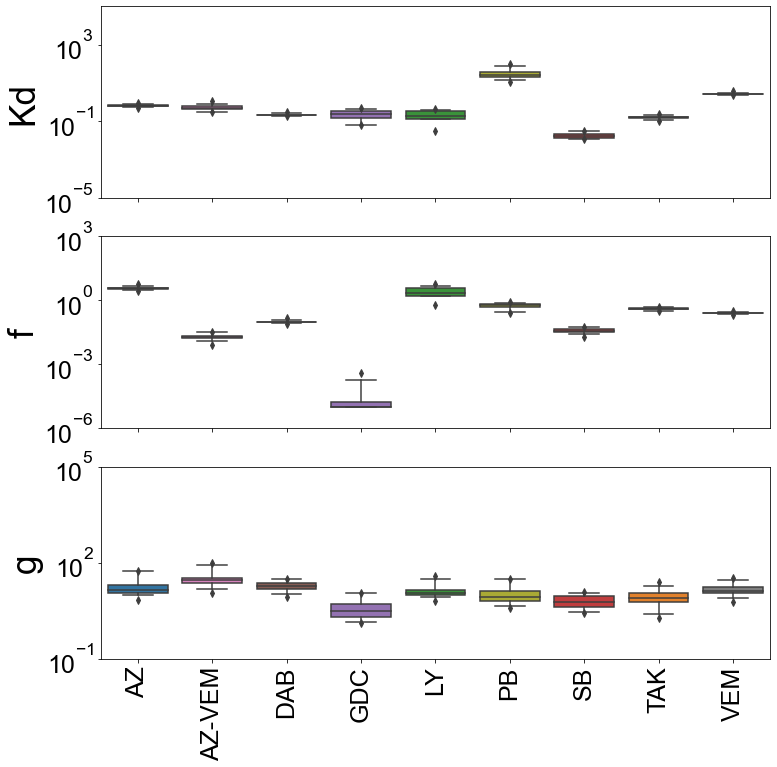

Supplement: Source code 1. — Contents include a folder that includes Mathematica notebooks that derive the analytic results. Contents also include a folder that includes Python notebooks that reproduce all of the numerical results. [file elife-82739-code1.zip › Supplementary_code/RAF_PA_Mechanisms/subModel_plots/28pars_normtp1_parameter_Kd_f_g.png]

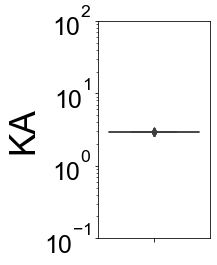

Supplement: Source code 1. — Contents include a folder that includes Mathematica notebooks that derive the analytic results. Contents also include a folder that includes Python notebooks that reproduce all of the numerical results. [file elife-82739-code1.zip › Supplementary_code/RAF_PA_Mechanisms/subModel_plots/28pars_parameter_KA.png]

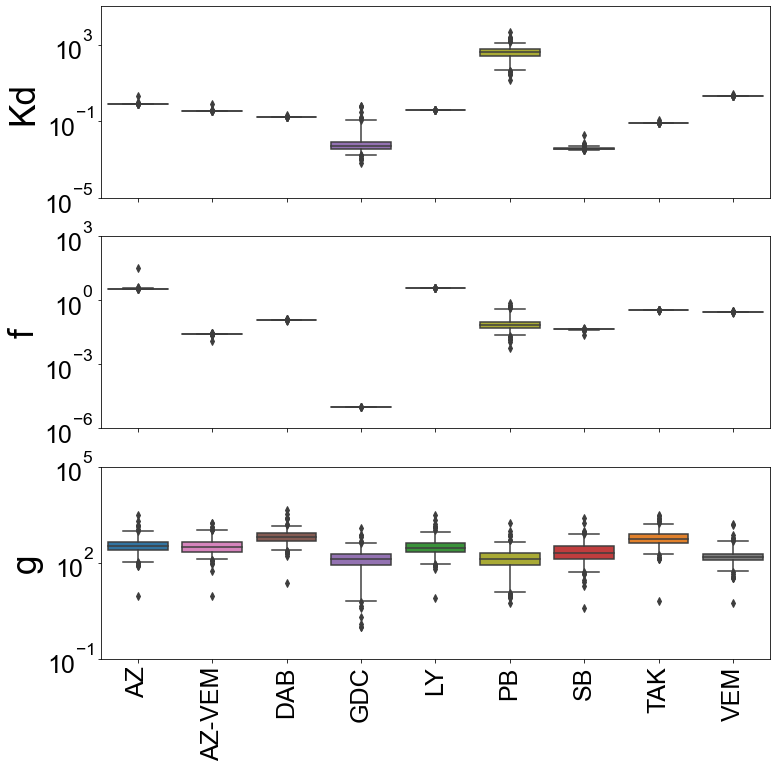

Supplement: Source code 1. — Contents include a folder that includes Mathematica notebooks that derive the analytic results. Contents also include a folder that includes Python notebooks that reproduce all of the numerical results. [file elife-82739-code1.zip › Supplementary_code/RAF_PA_Mechanisms/subModel_plots/28pars_parameter_Kd_f_g.png]

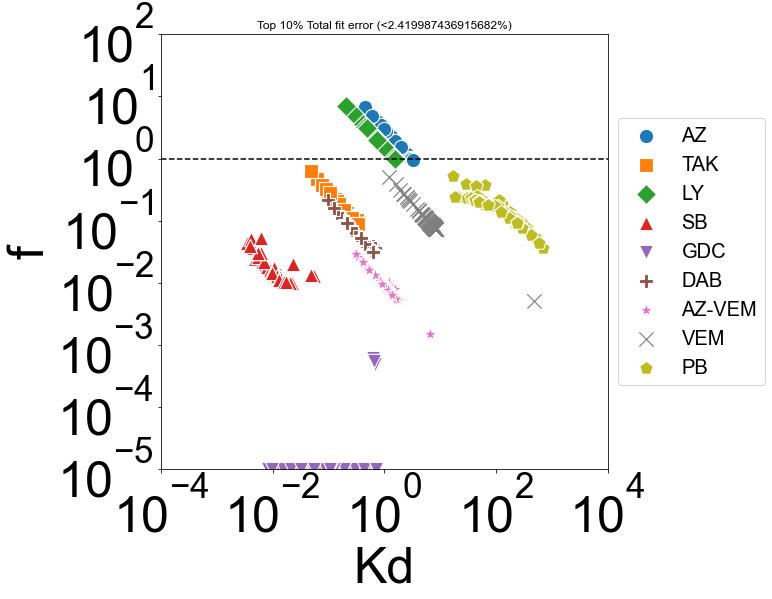

Supplement: Source code 1. — Contents include a folder that includes Mathematica notebooks that derive the analytic results. Contents also include a folder that includes Python notebooks that reproduce all of the numerical results. [file elife-82739-code1.zip › Supplementary_code/RAF_PA_Mechanisms/subModel_plots/30pars_Kd_vs_f.png]

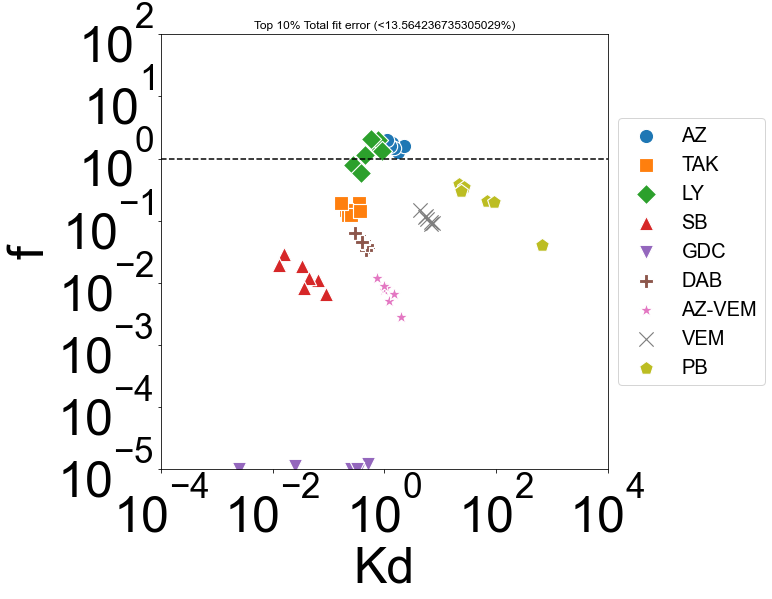

Supplement: Source code 1. — Contents include a folder that includes Mathematica notebooks that derive the analytic results. Contents also include a folder that includes Python notebooks that reproduce all of the numerical results. [file elife-82739-code1.zip › Supplementary_code/RAF_PA_Mechanisms/subModel_plots/30pars_normtp1_Kd_vs_f.png]

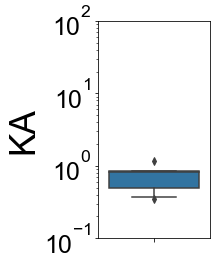

Supplement: Source code 1. — Contents include a folder that includes Mathematica notebooks that derive the analytic results. Contents also include a folder that includes Python notebooks that reproduce all of the numerical results. [file elife-82739-code1.zip › Supplementary_code/RAF_PA_Mechanisms/subModel_plots/30pars_normtp1_parameter_KA.png]

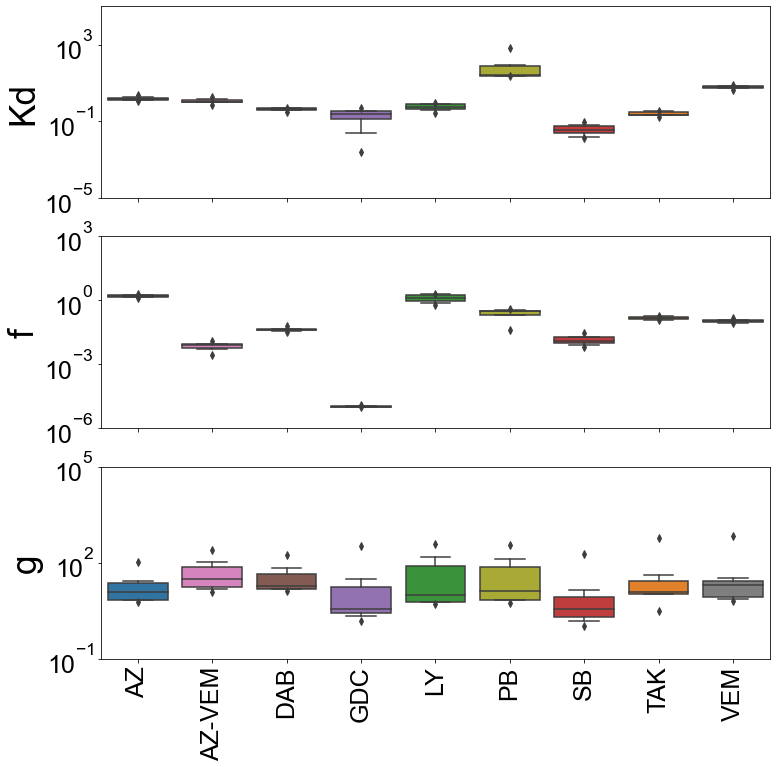

Supplement: Source code 1. — Contents include a folder that includes Mathematica notebooks that derive the analytic results. Contents also include a folder that includes Python notebooks that reproduce all of the numerical results. [file elife-82739-code1.zip › Supplementary_code/RAF_PA_Mechanisms/subModel_plots/30pars_normtp1_parameter_Kd_f_g.png]

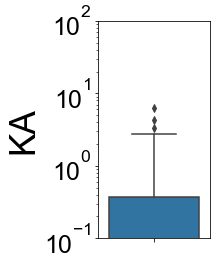

Supplement: Source code 1. — Contents include a folder that includes Mathematica notebooks that derive the analytic results. Contents also include a folder that includes Python notebooks that reproduce all of the numerical results. [file elife-82739-code1.zip › Supplementary_code/RAF_PA_Mechanisms/subModel_plots/30pars_parameter_KA.png]

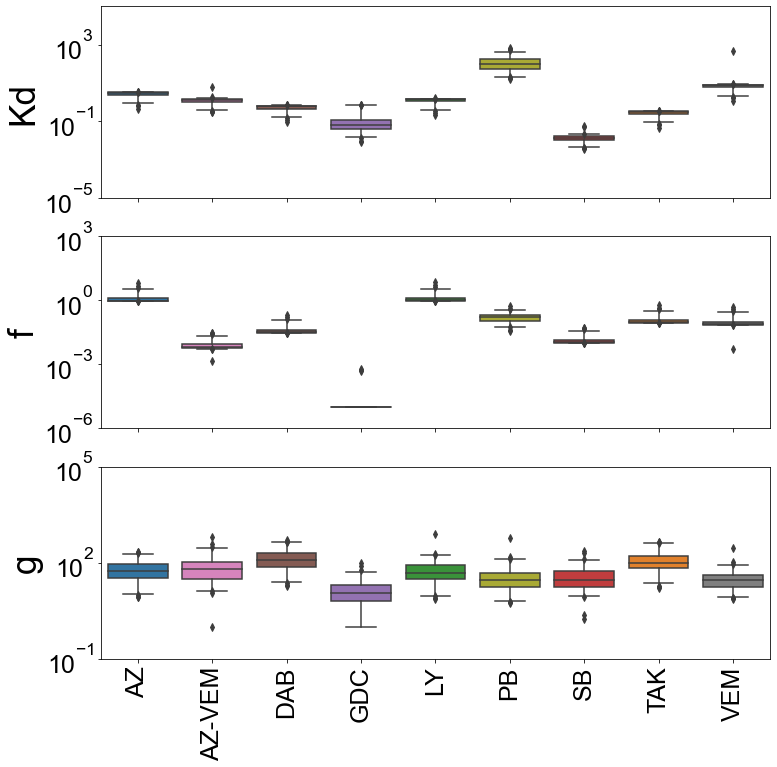

Supplement: Source code 1. — Contents include a folder that includes Mathematica notebooks that derive the analytic results. Contents also include a folder that includes Python notebooks that reproduce all of the numerical results. [file elife-82739-code1.zip › Supplementary_code/RAF_PA_Mechanisms/subModel_plots/30pars_parameter_Kd_f_g.png]

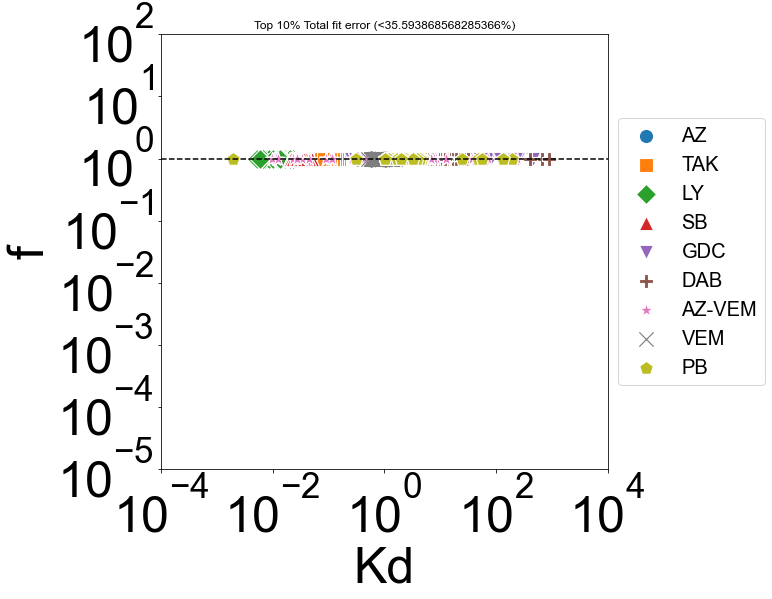

Supplement: Source code 1. — Contents include a folder that includes Mathematica notebooks that derive the analytic results. Contents also include a folder that includes Python notebooks that reproduce all of the numerical results. [file elife-82739-code1.zip › Supplementary_code/RAF_PA_Mechanisms/subModel_plots/CA_Kd_vs_f.png]

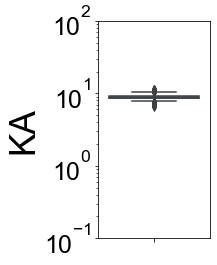

Supplement: Source code 1. — Contents include a folder that includes Mathematica notebooks that derive the analytic results. Contents also include a folder that includes Python notebooks that reproduce all of the numerical results. [file elife-82739-code1.zip › Supplementary_code/RAF_PA_Mechanisms/subModel_plots/CA_parameter_KA.png]

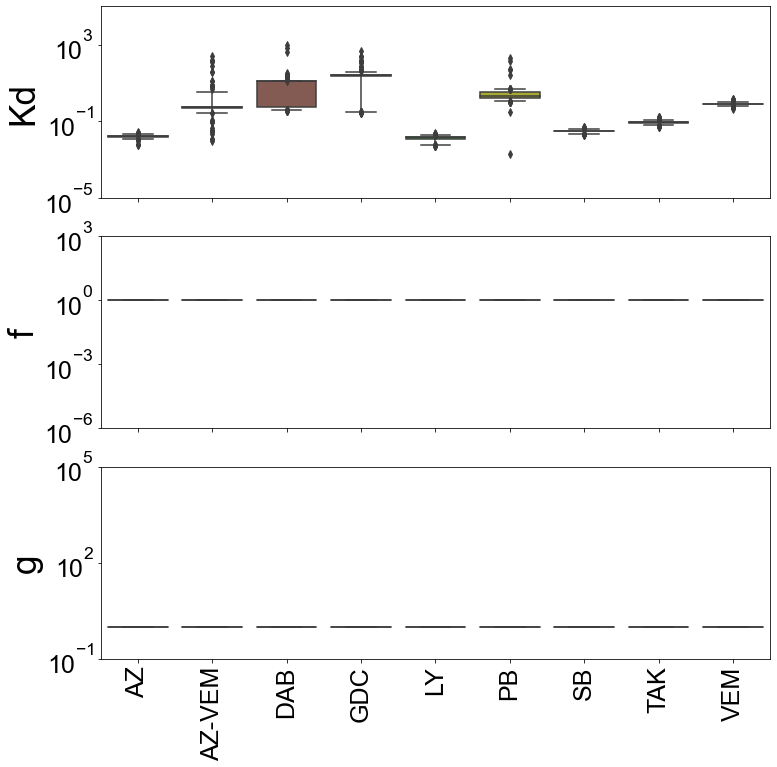

Supplement: Source code 1. — Contents include a folder that includes Mathematica notebooks that derive the analytic results. Contents also include a folder that includes Python notebooks that reproduce all of the numerical results. [file elife-82739-code1.zip › Supplementary_code/RAF_PA_Mechanisms/subModel_plots/CA_parameter_Kd_f_g.png]

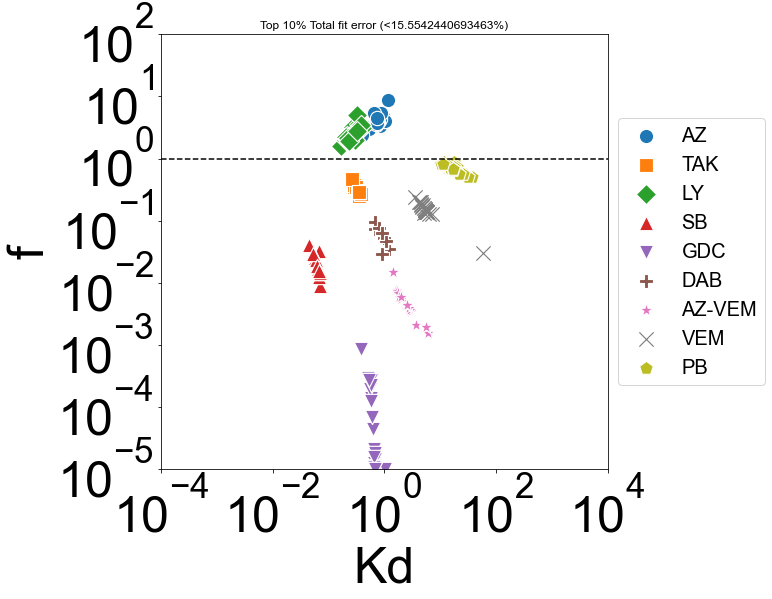

Supplement: Source code 1. — Contents include a folder that includes Mathematica notebooks that derive the analytic results. Contents also include a folder that includes Python notebooks that reproduce all of the numerical results. [file elife-82739-code1.zip › Supplementary_code/RAF_PA_Mechanisms/subModel_plots/CADP_Kd_vs_f.png]

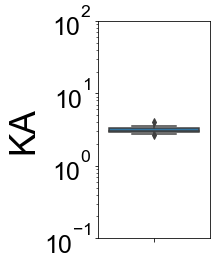

Supplement: Source code 1. — Contents include a folder that includes Mathematica notebooks that derive the analytic results. Contents also include a folder that includes Python notebooks that reproduce all of the numerical results. [file elife-82739-code1.zip › Supplementary_code/RAF_PA_Mechanisms/subModel_plots/CADP_parameter_KA.png]

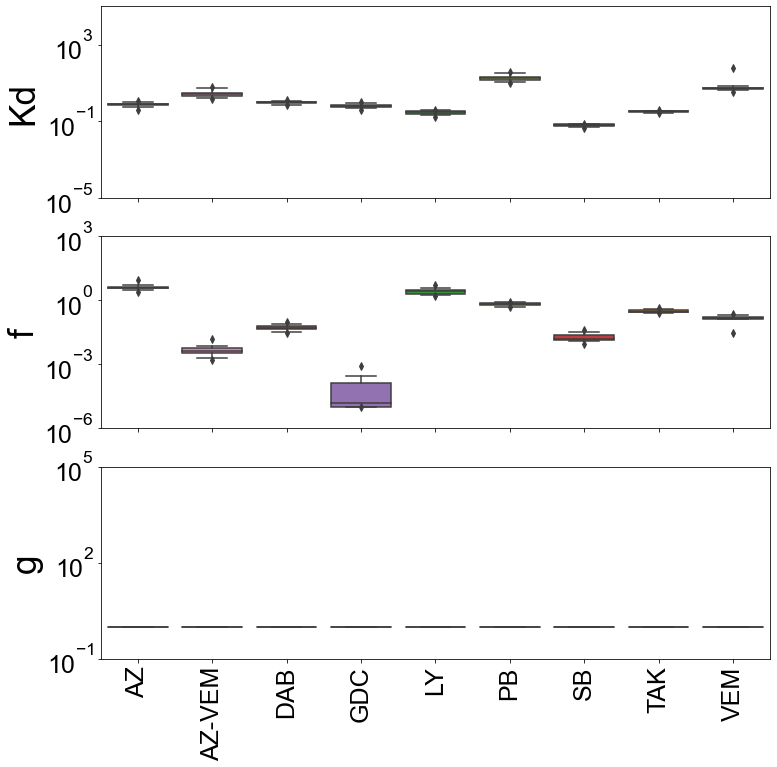

Supplement: Source code 1. — Contents include a folder that includes Mathematica notebooks that derive the analytic results. Contents also include a folder that includes Python notebooks that reproduce all of the numerical results. [file elife-82739-code1.zip › Supplementary_code/RAF_PA_Mechanisms/subModel_plots/CADP_parameter_Kd_f_g.png]

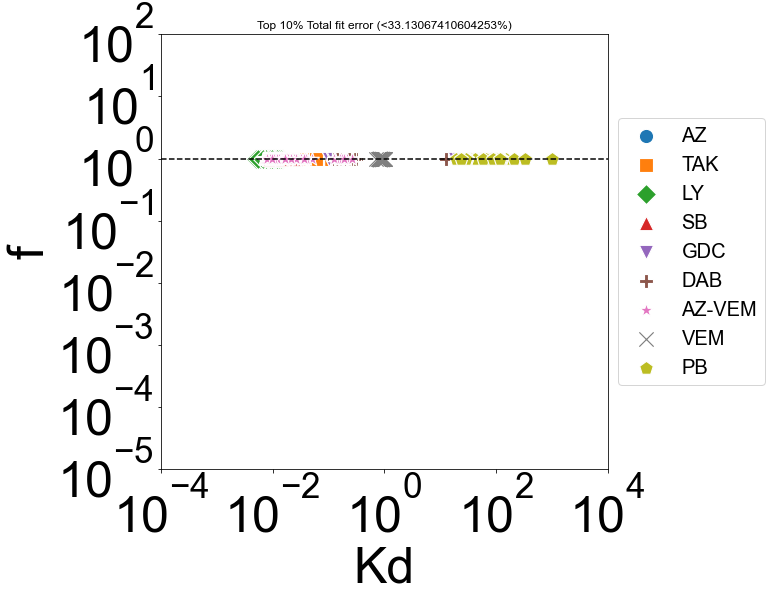

Supplement: Source code 1. — Contents include a folder that includes Mathematica notebooks that derive the analytic results. Contents also include a folder that includes Python notebooks that reproduce all of the numerical results. [file elife-82739-code1.zip › Supplementary_code/RAF_PA_Mechanisms/subModel_plots/CANC_Kd_vs_f.png]

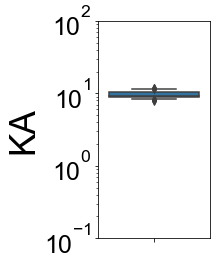

Supplement: Source code 1. — Contents include a folder that includes Mathematica notebooks that derive the analytic results. Contents also include a folder that includes Python notebooks that reproduce all of the numerical results. [file elife-82739-code1.zip › Supplementary_code/RAF_PA_Mechanisms/subModel_plots/CANC_parameter_KA.png]

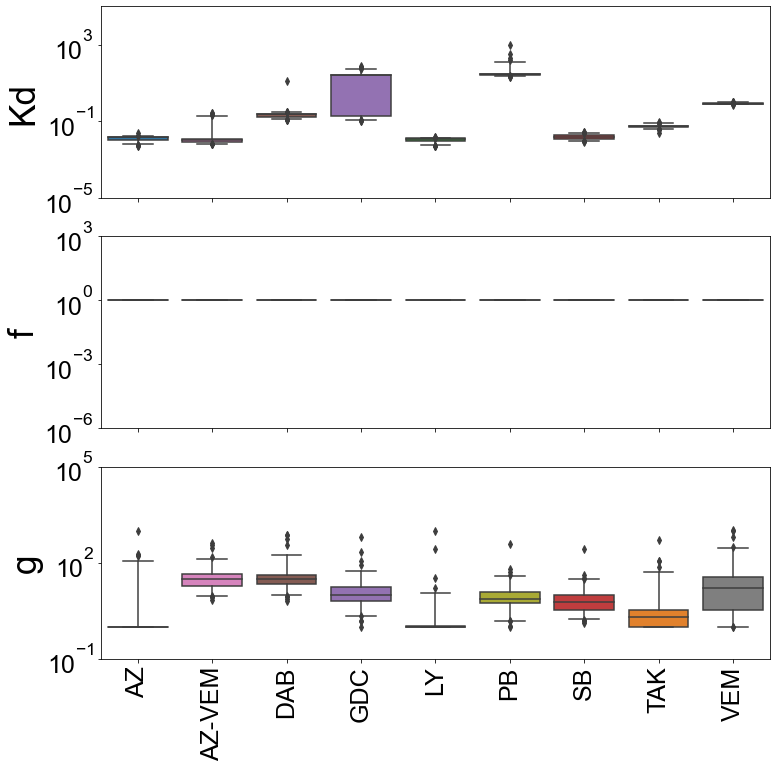

Supplement: Source code 1. — Contents include a folder that includes Mathematica notebooks that derive the analytic results. Contents also include a folder that includes Python notebooks that reproduce all of the numerical results. [file elife-82739-code1.zip › Supplementary_code/RAF_PA_Mechanisms/subModel_plots/CANC_parameter_Kd_f_g.png]

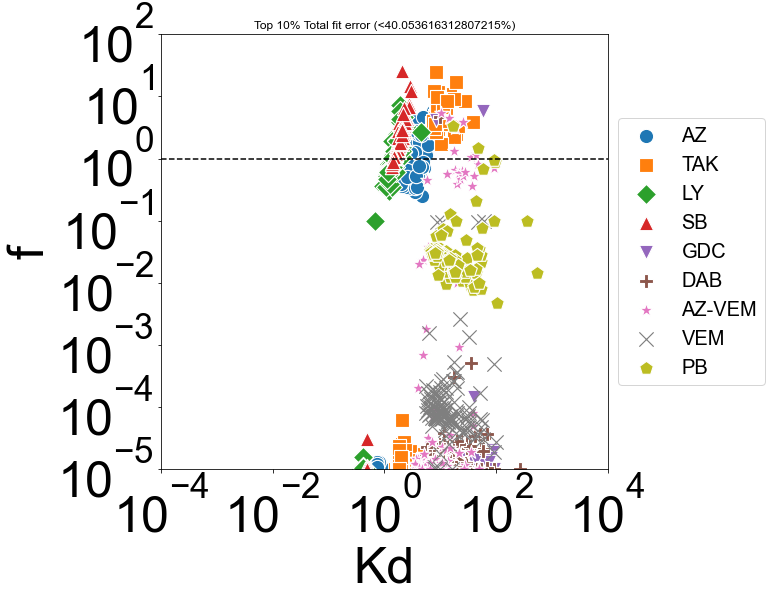

Supplement: Source code 1. — Contents include a folder that includes Mathematica notebooks that derive the analytic results. Contents also include a folder that includes Python notebooks that reproduce all of the numerical results. [file elife-82739-code1.zip › Supplementary_code/RAF_PA_Mechanisms/subModel_plots/DP_Kd_vs_f.png]

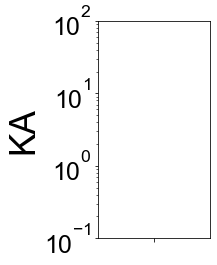

Supplement: Source code 1. — Contents include a folder that includes Mathematica notebooks that derive the analytic results. Contents also include a folder that includes Python notebooks that reproduce all of the numerical results. [file elife-82739-code1.zip › Supplementary_code/RAF_PA_Mechanisms/subModel_plots/DP_parameter_KA.png]

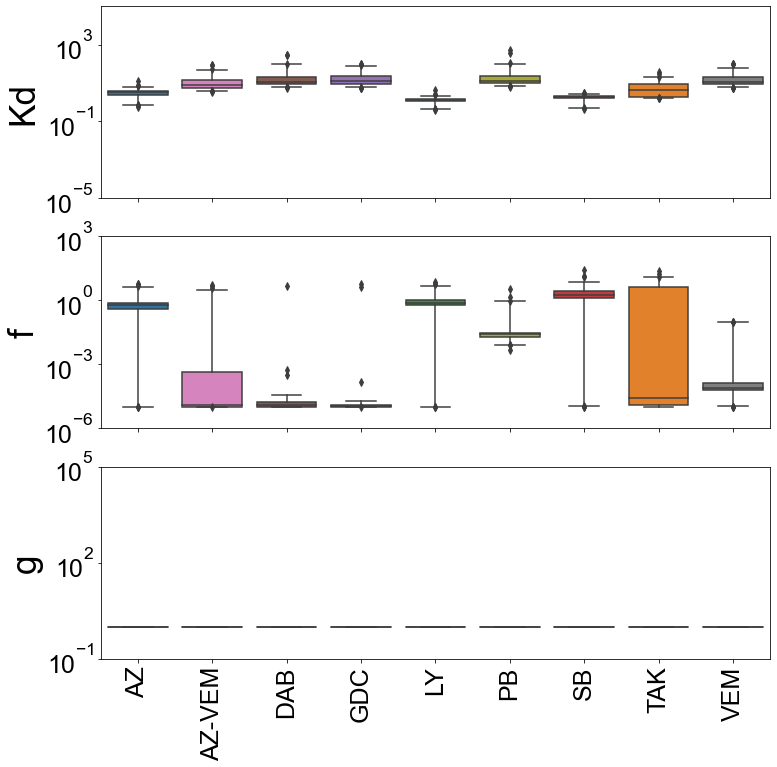

Supplement: Source code 1. — Contents include a folder that includes Mathematica notebooks that derive the analytic results. Contents also include a folder that includes Python notebooks that reproduce all of the numerical results. [file elife-82739-code1.zip › Supplementary_code/RAF_PA_Mechanisms/subModel_plots/DP_parameter_Kd_f_g.png]

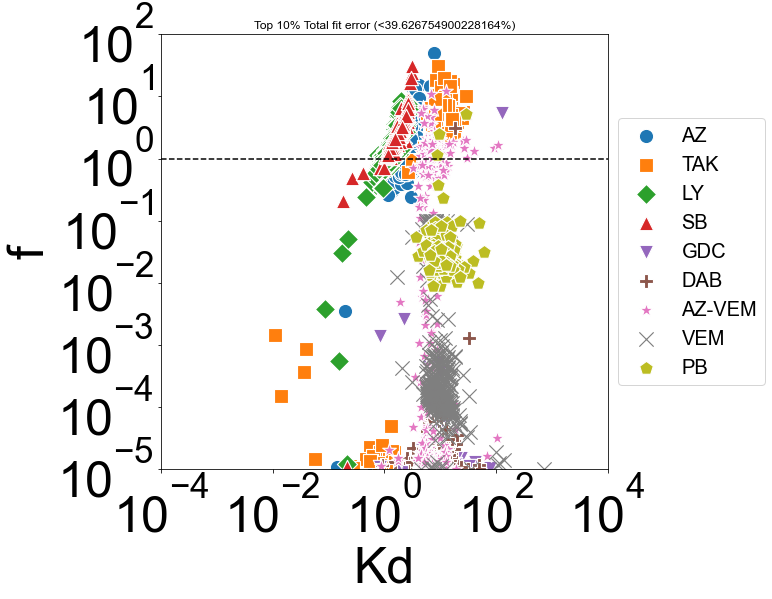

Supplement: Source code 1. — Contents include a folder that includes Mathematica notebooks that derive the analytic results. Contents also include a folder that includes Python notebooks that reproduce all of the numerical results. [file elife-82739-code1.zip › Supplementary_code/RAF_PA_Mechanisms/subModel_plots/DPNC_Kd_vs_f.png]

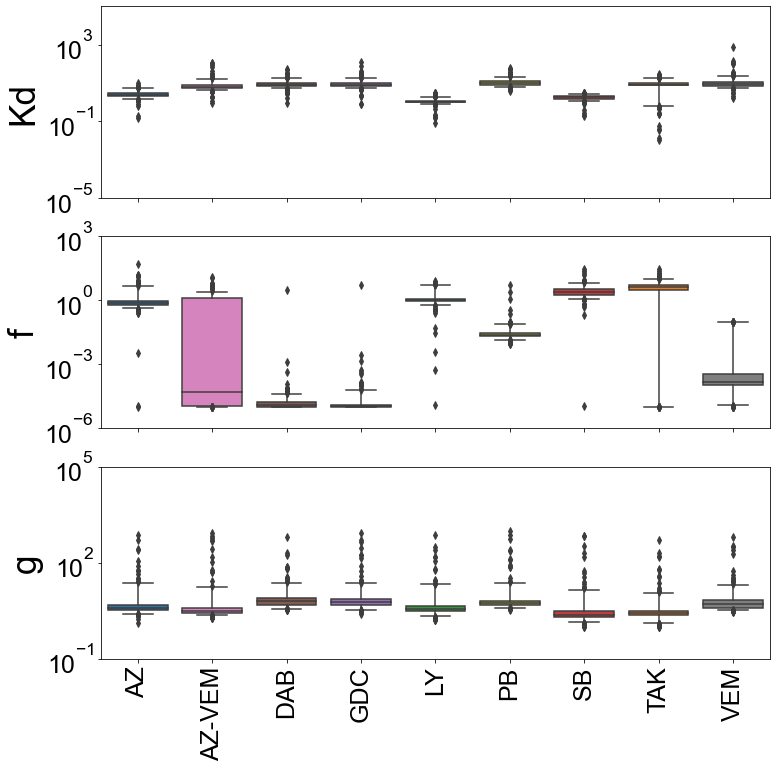

Supplement: Source code 1. — Contents include a folder that includes Mathematica notebooks that derive the analytic results. Contents also include a folder that includes Python notebooks that reproduce all of the numerical results. [file elife-82739-code1.zip › Supplementary_code/RAF_PA_Mechanisms/subModel_plots/DPNC_parameter_Kd_f_g.png]

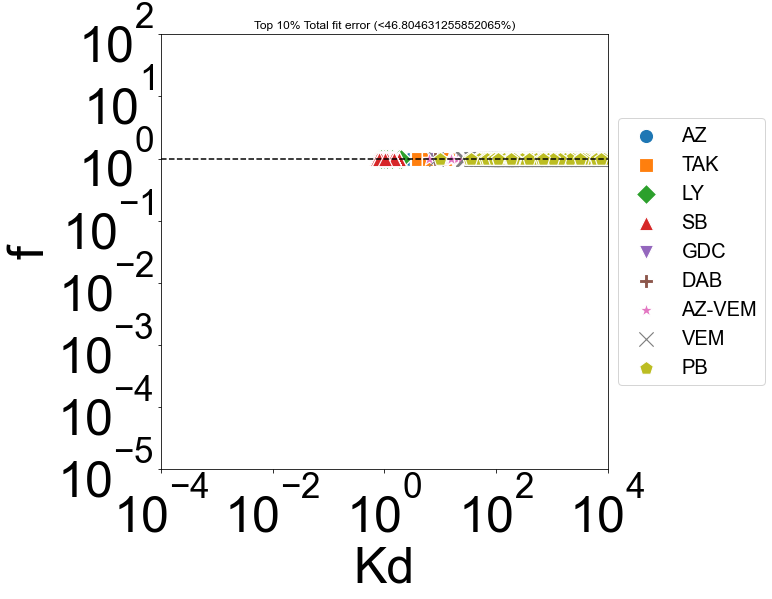

Supplement: Source code 1. — Contents include a folder that includes Mathematica notebooks that derive the analytic results. Contents also include a folder that includes Python notebooks that reproduce all of the numerical results. [file elife-82739-code1.zip › Supplementary_code/RAF_PA_Mechanisms/subModel_plots/NC_Kd_vs_f.png]

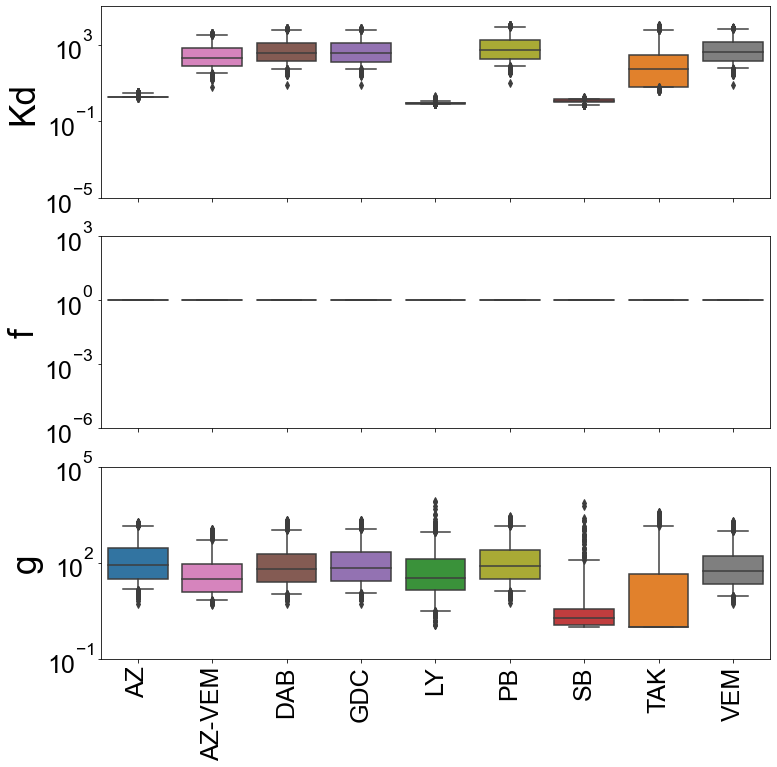

Supplement: Source code 1. — Contents include a folder that includes Mathematica notebooks that derive the analytic results. Contents also include a folder that includes Python notebooks that reproduce all of the numerical results. [file elife-82739-code1.zip › Supplementary_code/RAF_PA_Mechanisms/subModel_plots/NC_parameter_Kd_f_g.png]
